# Probing Local Coordination and Halide Miscibility in Single‑, Double‑, and Triple-Halide Perovskites Using EXAFS

**DOI:** 10.1021/acs.jpcc.6c03620

**Published:** 2026-08-05

**Authors:** Sonia S. Mulgund, Esther Y.-H. Hung, Leslie Bostwick, Ashley Galbraith, Owen M. Romberg, Justus Just, Rebecca A. Belisle

**Affiliations:** † Department of Physics and Astronomy, 8456 Wellesley College, Wellesley, Massachusetts 02481, United States; ‡ MAX IV Laboratory, Lund University, 22484 Lund, Sweden; § 10647 Olin College of Engineering, Needham, Massachusetts 02492, United States; ∥ Department of Chemistry, Wellesley College, Wellesley, Massachusetts 02481, United States

## Abstract

Lead-halide perovskites are a promising material platform as semiconductors in next-generation solar cells because of their solution processability, defect tolerance, and tunable optoelectronic properties. Mixed iodide–bromide perovskite compositions are attractive as wide-bandgap absorbers but suffer from significant operational instabilities. Incorporation of chloride to form triple-halide perovskites has been shown to improve both stability and performance; however, the extent of halide miscibility and chloride incorporation remains poorly understood. While bulk metrics such as diffraction-derived lattice parameters and optical bandgaps can confirm single-phase behavior, they do not establish homogeneous mixing on the halide site. Here, we use cryogenic X-ray absorption spectroscopy (XAS) to directly probe local lead-halide coordination across single-, double-, and triple-halide perovskite compositions. We show the formation of a single-phase triple-halide perovskite whose miscibility is mediated by bromide content. We identify signatures of halide mixing from the Pb L_3_-edge EXAFS of mixed double- and triple-halide perovskites using both quantitative fits and Cauchy wavelet transforms. Finally, using wavelet transforms of the Br K-edge EXAFS, we demonstrate halide intermixing on the single PbX_6_ octahedron level, exploiting forward-scattering amplified third-shell halide–bromide interactions. These results are a step forward in the understanding of local structure that is required to fully describe and optimize halide incorporation for novel perovskite compositions.

## Graphical abstract

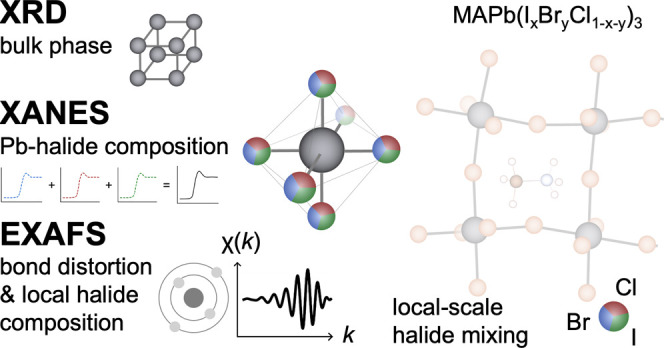

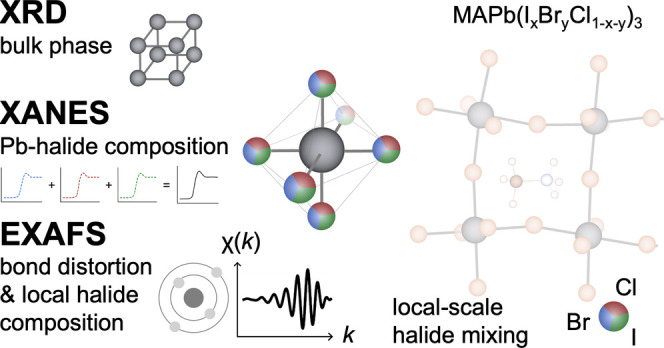

## Introduction

Lead-halide perovskites have rapidly established themselves as leading semiconductor candidates for next-generation photovoltaics, where reported power conversion efficiencies (PCEs) have exceeded 27% for single-junction devices^1^ and are approaching 35% for perovskite-silicon-tandem devices.^2^ The greater efficiencies achievable in tandem and multijunction devices are enabled by the facile tunability of perovskites through halide composition control.^3–7^ That tunability enables development of wide-bandgap perovskites (*E*_g_ > 2 eV), which have been operationalized in all-perovskite and perovskite-on-silicon multijunction devices.^2,5,8,9^ However, many wide-bandgap perovskites, typically combining iodide and bromide to access bandgaps up to approximately 2.4 eV, suffer from halide segregation that results in structural and electronic disorder and significant nonradiative losses.^7,10–13^ Photoinduced halide segregation is a reversible phenomenon where, upon illumination, halide migration results in the formation of temporary iodide-rich and bromide-rich regions of distinct bandgaps and lattice spacings.^14,15^ The exact mechanism underlying photoinduced halide segregation is debated, but whether induced by thermodynamic demixing,^16^ polaron-related lattice deformation,^17,18^ or photochemical halide oxidation,^19^ this detrimental phenomenon relies on halide migration through the perovskite lattice, and it is as such accelerated by preexisting defects and structural disorder.^20–22^

Thus, in order to mitigate photoinduced halide segregation and improve the performance of current wide-bandgap perovskites, a variety of approaches have been proposed, including chloride addition to direct crystallization pathway and promote increased grain size and quality.^6,11,23–25^ Chloride processing has been extensively studied for its role in improving crystallinity,^6,23–27^ photostability,^27^ and electronic performance^6,23,28^ of iodide-bromide perovskites by directing crystallization,^25^ filling lattice vacancies,^11^ and deactivating trap states.^11^ When chloride fraction is further increased, in addition to these dopant effects, chloride can incorporate into the bulk lattice alongside iodide and bromide to form a triple-halide perovskite phase, as first demonstrated by McGehee and co-workers—a new compositional variable that further raises the accessible bandgap to 3 eV while maintaining the beneficial structural and electronic properties from lower fractions of chloride incorporation.^5,7,19,29^ In multijunction devices, such triple-halide perovskites have the benefit of both a widened accessible bandgap range and improved stability to halide segregation.^5,30–32^

While bulk characterization metrics such as optical bandgap and the average crystal structure point toward near-stoichiometric chloride incorporation,^5^ the question remains whether chloride is truly incorporating into the perovskite, and if so to what extent and how homogeneously. In mixed iodide-bromide perovskites, the solid-state miscibility has been documented by X-ray diffraction (XRD) and spectroscopic methods as well as theoretical modeling, showing lattice spacing and bandgap to change proportionally with halide ratio.^3,33,34^ Signatures of phase segregation in double-halide materials are also well-understood, both from the same structural and optical methods, and by a suite of nanoscale imaging and elemental analysis techniques.^10,15,35–40^ For example, a significant miscibility gap has also been documented for MAPb­(Cl_*x*_I_1–*x*_)_3_ with chloride content as low as 3%, and many studies of chloride processing in iodide-rich perovskites suggest that it largely leaves the film during annealing.^25,41^ These results make quantification of chloride incorporation challenging. The Goldschmidt tolerance factor, *t*, is the classical geometric descriptor used to predict perovskite formability and degree of framework distortion for a given ABX_3_ composition, based solely on the constituent ionic radii.^42^ While a useful indicator of average symmetry, it treats the ions as hard spheres and so does not capture the covalent character of the Pb-X bond (which varies with halide identity), or the octahedral tilting and local distortions induced by hydrogen bonding between the organic cation and the halide sublattice—effects that are often unresolvable by XRD.^43^ These complexities mean a detailed understanding of miscibility in mixed-halide perovskites is still missing.

Thus, in order to fully describe miscibility and halide incorporation in triple-halide perovskites, both bulk metrics (i.e., lattice spacing and bandgap) and more local descriptions of the coordination environment are required. Such a description should not only show chloride content in the perovskite film, but also that the chloride is incorporated into the perovskite lattice itself, and that a single homogeneous phase has formed. X-ray absorption spectroscopy (XAS) is a powerful technique for this task, providing a view of the average coordination environment (up to 10 Å) of a given element in a material. When combined with XRD lattice spacing, both local geometry and long-range structure are illuminated. XAS has already been applied to study lead-halide perovskites toward a variety of goals: bond dynamics and temperature/pressure induced phase transitions,^44–49^ precursor evolution and film crystallization,^25,50–54^ and local coordination of formed materials.^24,55–60^ Here, we apply XAS to study a range of perovskite compositions, from single- to triple-halide, to confirm halide incorporation and determine EXAFS signatures of mixing. Our study moves toward a deeper structural understanding of triple-halide perovskites, thus enabling a more informed design of wide-bandgap absorbers in tandem solar cells.

## Experimental Methods

### Perovskite Fabrication

Perovskite thin-film samples were solution-processed on UV-ozone-cleaned polyamide substrates in a dry N_2_ environment. 1 M solutions of CH_3_NH_3_Pb­(I_*x*_Br_*y*_Cl_1‑*x*‑y_)_3_ were prepared by first making 1 M solutions of CH_3_NH_3_PbI_3_, CH_3_NH_3_PbBr_3_, and CH_3_NH_3_PbCl_3_ (PbI_2_, TCI; PbBr_2_, TCI; PbCl_2_, TCI; CH_3_NH_3_I, Greatcell Chemicals; CH_3_NH_3_Br, Greatcell Chemicals; CH_3_NH_3_Cl, Greatcell Chemicals) in anhydrous DMSO solvent (Sigma-Aldrich) and then combining these solutions in the appropriate volumetric ratios. All solutions were prepared in a N_2_ glovebox 24 h before spin-coating and filtered through a 0.2 μm PVDF filter before spin coating. For each sample, 30 μL of solution was converted into perovskite using the following process: 40 s of spinning at 6000 rpm; 30 s of immersion in anhydrous anisole with slight agitation; and 30 s of spinning at 6000 rpm to dry the film. All samples were then annealed at 100 °C for 30 min. Separate samples were fabricated for XRD and XAS measurements.

### X-ray Diffraction (XRD) Measurements

XRD data were collected in grazing incidence geometry at beamline 11–3 of the Stanford Synchrotron Radiation Lightsource. Two-dimensional scattering was collected with monochromatic 12.7 keV X-rays and recorded on a Rayonix MX-225 detector measuring 225 × 225 mm^2^. Samples were measured in a chamber under flowing helium at room temperature. All 2D images were calibrated with a LaB_6_ standard to determine experimental geometry. GSAS-II,^61^ pyFAI^62^ and pygix^63^ python packages were used for data analysis (complete methods included in the SI).

### X-ray Absorption Spectroscopy (XAS) Measurements

XAS data were collected in transmission mode at the Balder beamline of the MAX IV Laboratory 3 GeV synchrotron using a Si(111) double crystal monochromator with fixed exit motion and vertical beam stabilization. To achieve an edge step allowing for high quality transmission mode measurements of thin-films, samples (25 × 25 mm^2^) coated on polyimide substrate were cut into strips and stacked on top of each other. Samples were combined into stacks of 8, except for MAPb­(Br_0.6_Cl_0.4_)_3_, which was a stack of 6. Transmission was measured through this stack under a shallow incidence angle of approximately 5–10°. The beam energy was calibrated to the selenium K-edge at 12654 eV (maximum of first derivative of μd). Signals of both incident (*I*_0_) and transmitted (*I*_*t*_) X-rays were detected using ionization chambers. During measurement, samples were cooled in a liquid helium cryostat to a temperature of 15–20 K. For each sample, 10–20 spectra were recorded and subsequently merged, using an acquisition time of 30s per EXAFS spectrum. Background subtraction and normalization of raw data was performed in the ATHENA software, and the ARTEMIS software with FEFF6 and ATOMS was used to model the processed EXAFS spectra.^64,65^ The wavelet transforms (Figures 3 and 5) were computed using the Cauchy method, implemented in LARCH.^66,67^

## Results and Discussion

There are many existing examples of mixed-halide perovskite systems, spanning bromide-chloride,^68^ bromide-iodide,^3^ and mixed-cation triple-halide perovskites.^5,29–31^ However, solution-processed single-cation triple-halide perovskites with significant chloride content remain largely unexplored. To reduce the complexity associated with mixed-cation disorder and enable clearer insight into halide miscibility, we focus on single-cation methylammonium lead halide perovskites, MAPb­(I_*x*_Br_*y*_Cl_1‑*x*‑y_)_3_.

To probe the crystallographic structure and phase purity of the mixed-halide thin films, we perform X-ray diffraction (XRD) measurements on as-cast thin films (Figure 1a). All films exhibit a reflection at *Q* ∼ 1Å with a strong preferential orientation in the out-of-plane direction (Figure 1c–f), assigned to the (100) cubic perovskite reflection. Relative to MAPbBr_3_ (dashed blue line), the 60% Br: 40% I and the 60% Br:40% Cl films show systematic shifts to lower and higher *Q*, indicating larger and smaller unit cells, respectively (*a* = 6.04 Å and *a* = 5.81 Å, Table S1). This trend is consistent with the larger ionic radius of I^–^ and the smaller ionic radius of Br^–^/Cl^–^ (Table S11), suggesting substantial halide mixing inside the perovskite lattice. The lack of substantial competing crystalline phases (e.g., PbI_2_) supports the existence of a crystalline majority (>90%) mixed-halide perovskite phase whose long-range compositional heterogeneity is within the width of the diffraction peaks.

**Figure 1:**
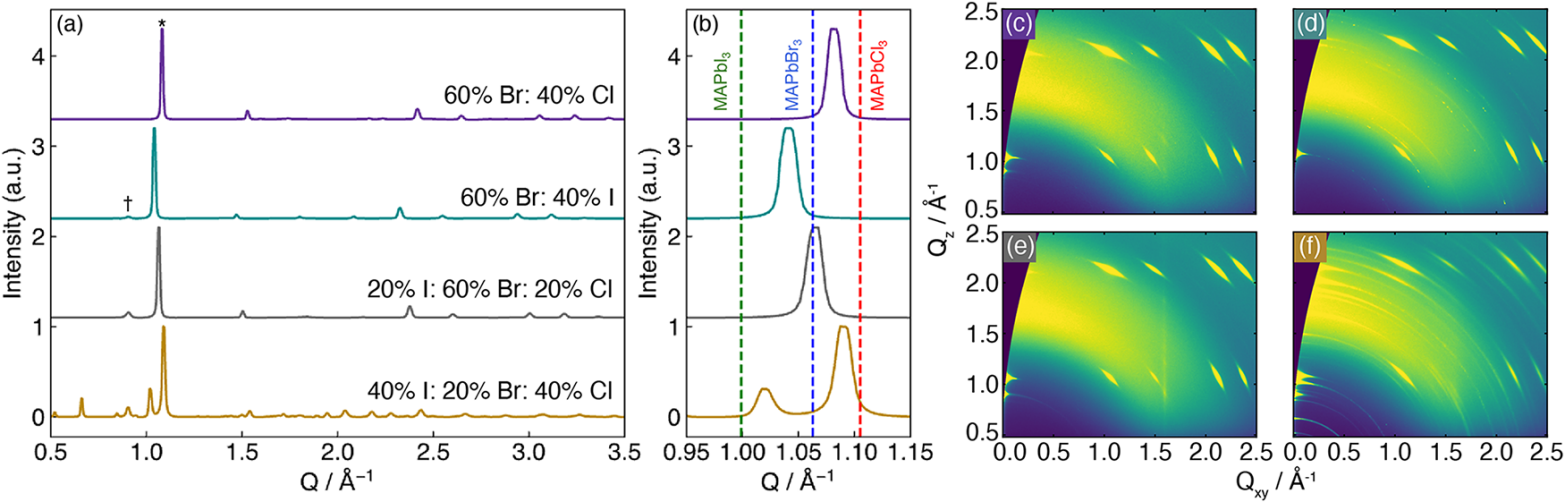
XRD patterns of mixed-halide perovskites. (a) Azimuthally integrated diffraction patterns of the mixed-halide perovskite thin-films, highlighting the cubic (100) (*) and PbI_2_ (†) reflections. (b) Enlargement of the region of the first perovskite reflection, showing single phase formation in all but the low Br content triple-halide perovskite. Dashed lines indicate the (100) peak center of the pure compositions MAPbCl_3_,^69^ MAPbBr_3_,^70^ and MAPbI_3_.^69^ (c)-(f) 2D XRD patterns in linear scale of the four mixed-halide samples with halide ratios: (c) 60% Br: 40% Cl, (d) 60% Br: 40% I, (e) 20% I: 60% Br: 20% Cl, (f) 40% I: 20% Br: 40% Cl.

XRD patterns for two triple-halide compositions, with low (20%) and high (60%) target Br^–^ content, highlight the role of composition in phase stability (Figure 1a, lower). At high Br^–^ content, a single (100) reflection with a peak width similar to the binary mixtures is observed (Table S1). The high Br content apparent triple-halide perovskite, MAPb­(I_0.2_Br_0.6_Cl_0.2_)_3_, shows a lattice spacing intermediate to that of the Br^–^/Cl^–^ and Br^–^/I^–^ perovskites (*a* = 5.91 Å), supporting the incorporation of all three halides into the bulk lattice and the formation of a single-phase perovskite. The similarity in peak center for the 60% Br triple-halide and MAPbBr_3_ highlights the challenge of inferring stoichiometry and compositional homogeneity from diffraction data alone for triple-halide compositions. In contrast to the 60% Br^–^ composition, the triple-halide film prepared from the 40% I: 20% Br: 40% Cl stoichiometry exhibits a clear splitting of the (100) reflection, consistent with phase segregation into two perovskite-like phases one Cl^–^-rich and one I^–^-rich. This suggests that the higher Br^–^ content promotes halide miscibility in the perovskite lattice, consistent with prior reported trends reported for mixed-cation and mechanochemically synthesized triple-halide perovskites.^5,29^ We also observe additional reflections attributable to unknown, non-3D-perovskite crystalline phases in the nominal MAPb­(I_0.4_Br_0.2_Cl_0.4_)_3_ composition.

The corresponding 2D-XRD patterns for each film (Figure 1c–f) reveal a perovskite structure that is highly textured independent of halide ratio, with all compositions showing a preferential orientation of the (n00) reflections along the *Q*_*z*_ direction. This consistent out-of-plane alignment suggests that the crystalline orientation is not strongly influenced by as-cast halide composition. The highest Cl^−^ content single-phase composition MAPb­(Br_0.6_Cl_0.4_)_3_ (Figure 1c) exhibits well-defined Bragg reflections, and has the lowest fitted perovskite peak width (Table S1)—consistent with known effects of Cl^–^ on perovskite crystallinity.^6^ In contrast, the phase-segregated nominal MAPb­(I_0.4_Br_0.2_Cl_0.4_)_3_ composition (Figure 1f) appears less sharply textured, with perovskite reflections which are more smeared radially.

These XRD results represent the long-range, average perovskite crystal structure. While they can verify single or segregated perovskite phase formation, they do not describe how the three halides interact with and accommodate one another, leaving open the possibility of compositional heterogeneity at a very local length scale. To describe that, local coordination as probed by XAS is a more appropriate tool.

Perovskite chemical composition influences long-range crystalline phase, short-range local coordination environment, and electronic orbital structure, so each of these should contain evidence of halide mixing. To probe these effects, we first employ XANES to investigate how halide composition modifies the electronic structure arising from the interactions between lead and halide electronic orbitals. Single-halide perovskite XANES *μ­(E)* spectra (Figure 2a) reveal distinct spectral features, indicating differences in electronic structure that reflect unique local chemical environments. Notably, the overall EXAFS oscillation amplitude is low despite measurement at 15–20 K, reflecting the significant static disorder in lead-halide perovskites compared to most crystalline materials at cryogenic temperatures where thermal/dynamic disorder is minimized. We study mixed-halide compositions using linear combination analysis (LCA) (Figure 2b–e), which approximates the Pb local environment as a superposition of that in MAPbI_3_, MAPbBr_3_, and MAPbCl_3_, consistent with a linear combination of atomic orbitals.^55^ However, a mixed-halide lattice is significantly distorted to accommodate the varying ionic radii.^3,34,71^ These changes in geometry influence how atomic orbitals combine to form the band structure of a mixed-halide perovskite; thus, the LCA fits (Figure 2b–e) provide only a crude indication of halide content in the absence of describing the intricacies of Pb-X interactions.

**Figure 2:**
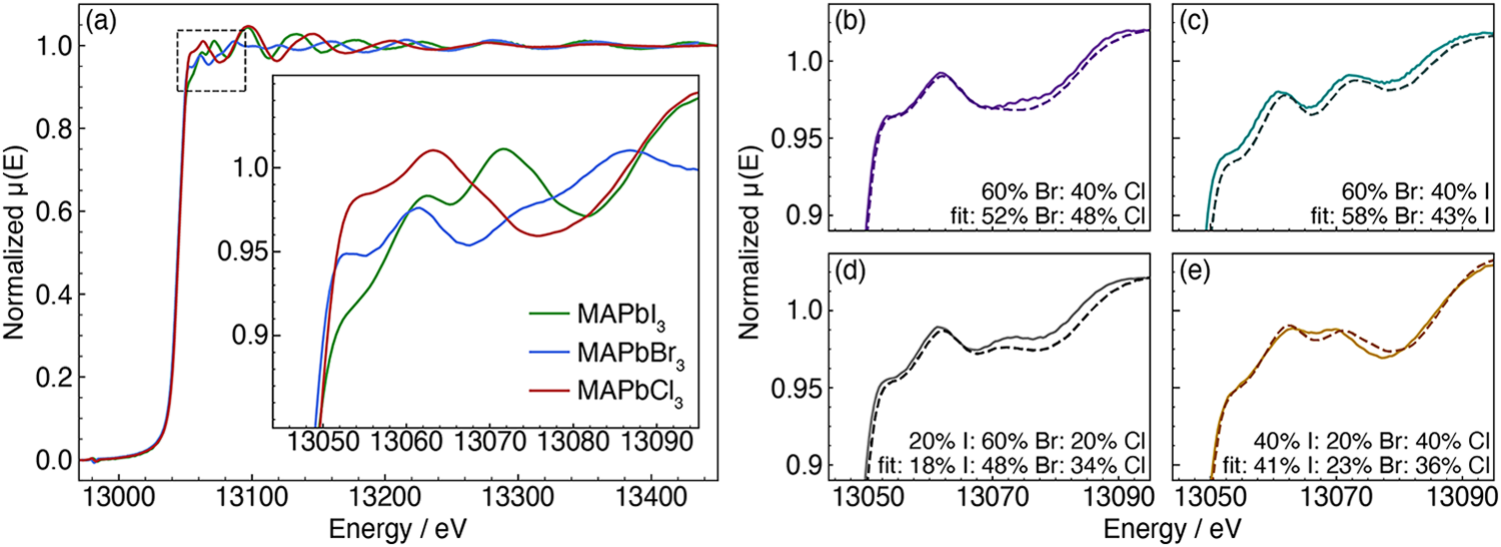
Normalized Pb L_3_-edge X-ray absorption near edge structure (XANES) spectra for all compositions, measured at cryogenic temperatures. (a) Normalized μ*(E)* with detail inset for single halide perovskites. (b–e) Normalized XANES *μ­(E)* for mixed-halide perovskites together with linear combination analysis (LCA) fits (dashed lines) for a quantitative estimate of halide coordination around Pb in comparison with the halide fractions for solution compositions.

Despite that limitation, linear combination of the single-halide perovskite XANES can reproduce all major spectral features for the mixed-halide compositions very well, demonstrating the presence of all three Pb-X bonds at significant fractions. There is no indication, for example, that either of the triple-halide samples completely lack Cl^–^ incorporation into the perovskite lattice. Moreover, the best-fit fractions resemble, within expected experimental uncertainties of approximately 5–10%, the solution stoichiometry. The largest discrepancy is for MAPb­(I_0.2_Br_0.6_Cl_0.2_)_3_, where the LCA fit indicates an effective stoichiometry of MAPb­(I_0.18_Br_0.48_Cl_0.34_)_3_, with substantially increased apparent Cl^–^ content and moderately reduced Br^–^ content compared with the solution composition. The LCA model is limited in its ability to capture the electronic structure of mixed-halide perovskite compositions, which deviate from a simple linear combination of single-halide orbitals. As a result, the elevated Cl^–^ fraction is unlikely to reflect a true increase in Cl^–^ content but instead represents an effective parameter in the linear combination fit that attempts to account for these more complex electronic and structural contributions. On the other hand, the LCA fits for the phase-segregated, nominal 20% Br^–^ triple-halide composition yield LCA fractions more closely resembling the solution stoichiometry. As identified in XRD, this composition likely forms distinct I^–^-rich and Cl^–^-rich crystalline phases, likely to each be less complex electronically than the single-phase triple-halide. The phase separation allows most bond distances and angles to relax closer to their single-halide values—thus making the linear combination assumptions more appropriate and enabling more accurate comparison of its XANES to the single-halide standards.^5,55^

Overall, the LCA results show that all halides contribute significantly to the measured XANES spectra at levels broadly consistent with the input solution stoichiometry. Thus, despite not providing a specific structural model, the Pb L_3_-edge XANES LCA demonstrates that all halides coordinate to Pb. This result, coupled with our observations of single perovskite phases in XRD, suggests that if this lead-halide coordination occurs in a crystalline phase, it must be in a mixed-halide perovskite phase. While small quantities of Cl^–^ have been shown to direct perovskite crystallization without incorporation into the bulk lattice,^25^ we now show based on the local environment of the Pb atom, instead of bulk metrics, that at higher Cl^–^ fractions it coordinates in the perovskite.

While XRD and XANES LCA show evidence of a triple-halide perovskite phase, a more direct local probe is needed to understand the extent of halide mixing and stoichiometric incorporation into the perovskite lattice. In contrast to XANES, EXAFS provides element-specific, local structural information by quantifying the identity, distances, and coordination numbers of atoms surrounding the absorber, typically out to approximately 8–10 Å. In lead-halide perovskites, this means that EXAFS can resolve the average PbX_6_ octahedral environment by identifying Pb-X nearest-neighbors along with tracking subtle changes in disorder. To minimize thermal disorder contributions to the EXAFS signal, we employ cryogenic EXAFS at 15 K at the Pb L_3_-edge (Figure 3a). All mixed, Br-containing halide data is cut off at the Br K-edge at 14374 eV, limiting χ*(k)* to 10 Å^–1^. The mixed-halide EXAFS χ*(k)* spectra (Figure 3a) reveal the presence of multiple frequency (*k*) components. In EXAFS, *k* is the wavevector of the photoelectron ejected from the absorbing atom (here, Pb). The backscattering amplitude and phase are characteristic of the neighboring atom and vary with *k* according to atomic number: lighter atoms generally backscatter at lower *k* and heavier atoms tend to contribute at higher *k*. Distinct backscattering atoms therefore contribute to χ*(k)* with different *k*-dependence (calculated amplitudes and phases for Pb-X single scattering paths shown in Figure S10). Accordingly, the mixed-halide data contain several superimposed components, indicating the presence of multiple distinct backscattering atoms (Cl, Br, I) around the absorbing Pb. To clarify these overlapping contributions to the EXAFS, we compute the Cauchy wavelet transform (WT). The WT describes the EXAFS oscillations as a superposition of wavelets, localized at a specific frequency and photoelectron wavenumber *k*, to yield a two-dimensional representation of the EXAFS signal, W­(*k*,R*), that simultaneously resolves contributions in *k* and R* (Figure 3b–h).^66^ As R* incorporates both the radial distance *R* and the scattering phase shift, which is individual for specific absorber-backscatterer combinations, its absolute value cannot be directly interpreted as a distance in real space. The distribution along the *k-*axis, as mentioned above, depends on the atomic number of neighboring atoms, allowing differentiation of atomic species at distances R* based on the *k*-dependent scattering amplitude. This allows direct visualization of how scattering contributions evolve in both domains. For the single-halide MAPbX_3_ compositions (Figure 3b–d), the main wavelet intensity feature, corresponding to the first shell halides, shifts toward higher R* and higher *k* as X varies from Cl to Br to I. This trend is consistent with the longer Pb-X bond distances associated with the increasing ionic radius going from lighter (Cl^–^) to heavier (I^–^) ions (Table S11) as well as the expected behavior that the *k*-dependent scattering amplitude favors higher *k* for heavier atoms.

**Figure 3:**
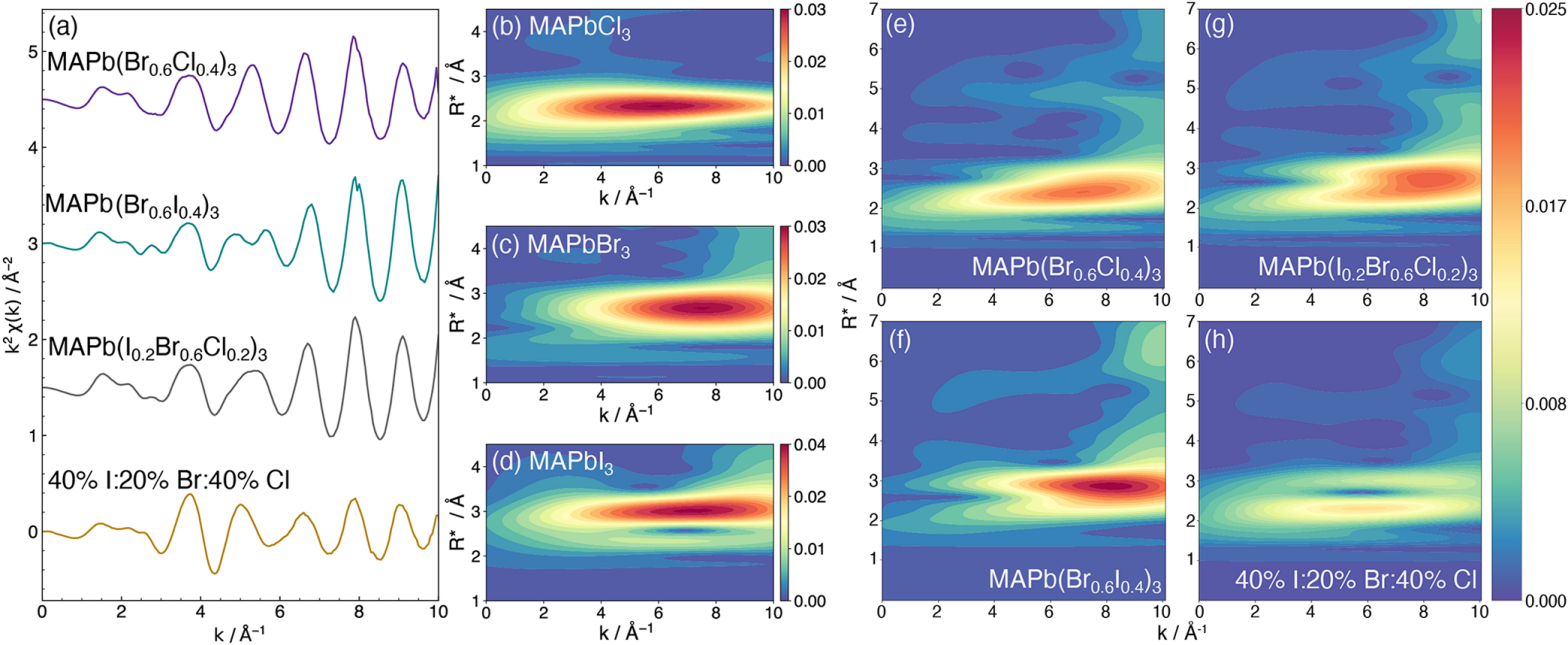
*k* ^*2*^ *-*weighted Pb L_3_—edge EXAFS and wavelet transforms thereof. (a) Pb L_3_ EXAFS χ*(k)* for the mixed-halide perovskite samples. Data is offset for visualization. (b–d) Cauchy wavelet transforms (WTs) W­(*k*,R*) for single halide perovskites in the range of the first Pb-X shell. (e–h) Cauchy WTs for the mixed-halide compositions.

A similar trend is observed in MAPb­(Br_0.6_X_0.4_)_3_, where the maximum wavelet intensity shifts toward higher R* and higher *k* when comparing X = Cl to X = I, indicating the presence of significant amounts of Cl^–^ in the structure in agreement to XANES. Across all mixed-halide compositions (Figure 3e–h), the high-intensity regions appear more broad along R* than for the single-halides, indicating an increased distribution of local Pb-X bond distances. In the triple-halide compositions, these high-intensity regions appear as distinct lobes, which overlap in MAPb­(I_0.2_Br_0.6_Cl_0.2_)_3_ (Figure 3g) but are separated in nominal MAPb­(I_0.4_Br_0.2_Cl_0.4_)_3_ (Figure 3h). This separation can reflect the reduced presence of intermediate-length Pb–Br bonds at lower Br content, consistent with the observed crystallographic phase-segregation. We note that these two-dimensional representations of the first Pb L_3_ coordination shell do not allow unequivocal distinction between single-phase and phase-segregated perovskite compositions, because the average Pb is still coordinated to all halides in both cases. However, they provide evidence that all three halides are present and coordinated to Pb in the perovskite lattice, consistent with XANES LCA.

To resolve distinct Pb-X contributions to the EXAFS, we next perform quantitative fitting of the EXAFS spectra. First shell fits are carried out in back-Fourier-transformed *q*-space using a PbX_6_ octahedral model, establishing benchmark Pb-X bond distances and disorder parameters for the single-halide compounds (Table S3). These fits show a systematic contraction of the Pb-X bond with decreasing halide ionic radius (Figure S8 and Table S3), with best-fit Pb-X distances ranging from 3.12 (X = I) to 2.73 Å (X = Cl), in agreement with published crystal structure data (Table S2).^3,33,34^ Of note, MAPbCl_3_ has a lower symmetry structure with four distinct Pb–Cl distances, which was taken into account in the fit. For MAPbI_3_, we additionally extended the fit to successfully include second shell Pb–Pb–Pb and multiple scattering contributions (Figure S9), validating the appropriateness of the orthorhombic perovskite input model.

Among the single-halide compositions, the Pb-X scattering path in MAPbBr_3_ exhibits the largest fitted mean-squared relative displacement (MRSD σ^2^, which describes the distribution of distances between the absorbing atom and a specific backscatterer: here, in Pb-X), indicating greater disorder than in the other single-halide compositions. At the measurement temperature (15–20 K), this disorder is predominantly static (i.e., structural rather than thermal). In single-halide MAPbX_3_, this static disorder encompasses octahedral tilting and MA^+^ orientational effects, which are coupled, especially at low temperature. The Pb L_3_-edge EXAFS does not directly probe MA^+^ orientation—the organic cation contains no heavy backscattering atoms—but it is sensitive to the correlated PbX_6_ framework response, including octahedral tilt and distortions. The larger σ^2^ for MAPbBr_3_ compared with the other single-halide compositions is consistent with reports elsewhere,^45,48,58,71,72^ which indicates more local static disorder compared with the iodide- or chloride-based perovskites. However, the limited energy range of the data due to the nearby Br K edge at 13474 eV limits the accuracy of this value.

Extending this analysis to mixed-halide compositions, quantitative EXAFS fits are performed in *q*-space using *ab initio-*calculated scattering amplitudes and phases, with the corresponding *R*-space results shown in Figure 4 (fitted parameters in Table S4). The fit (dashed black lines) represents the magnitude of the coherent complex sum of the individual scattering path contributions (colored curves) for distinct backscattering halide atoms within the first coordination shell of Pb, here the PbX_6_ octahedron (Figure 4d). The first shell model uses a single average Pb-X distance for each halide species and thus does not explicitly resolve the distinct X environments potentially present in the low-temperature orthorhombic structure. However, dual-halide perovskites have been shown experimentally to favor cubic structures at lower temperatures than their single-halide parent compositions,^3,73,74^ with our mixed-halide perovskites all being cubic at room temperature as shown in XRD (Figure 1). Likewise, computational studies of halide mixing suggest that compositional disorder and strain can reduce octahedral tilting, rendering the local structure in these materials more cubic-like than a single-halide perovskite.^71,74^ Under these conditions, the first shell environment is likely well-modeled by single Pb-X bond lengths for each X.

**Figure 4:**
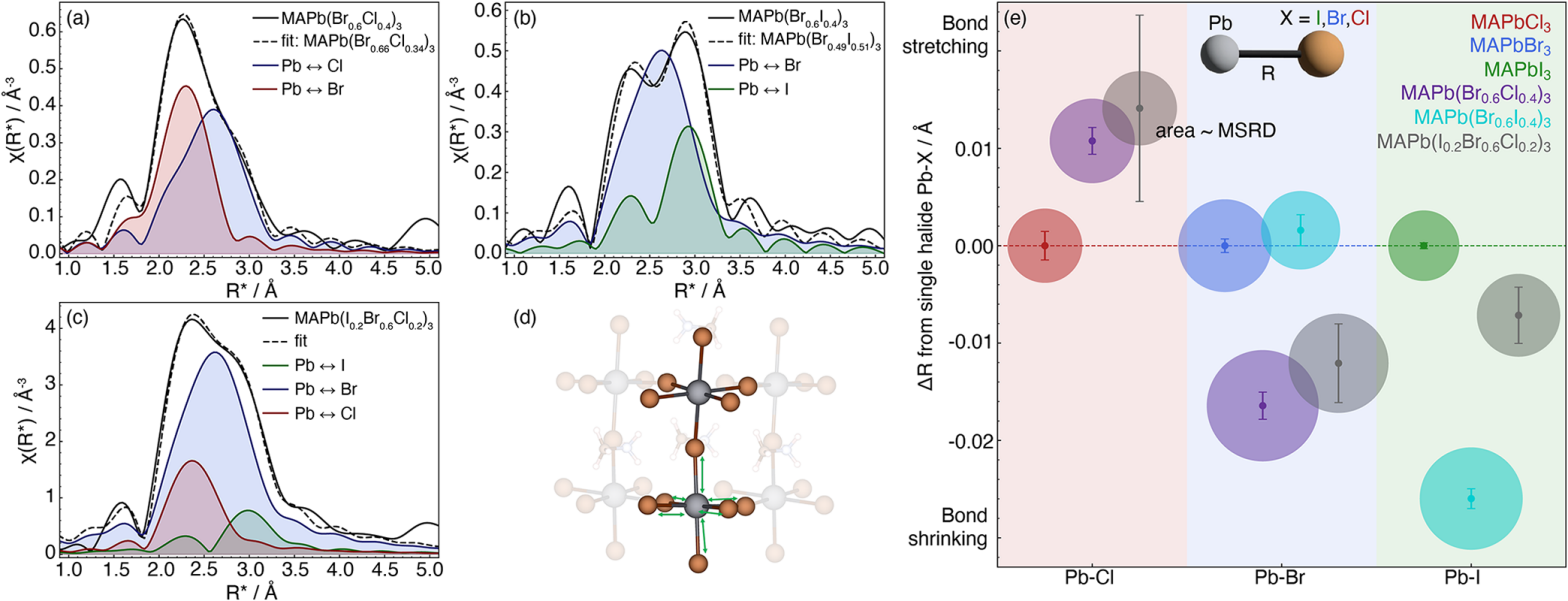
Local structure of PbX_6_ octahedra as derived from quantitative EXAFS analysis. (a–c) Magnitudes of the Fourier transform of Pb L_3_-edge EXAFS fits into R*-space for the mixed-halide samples. Solid black lines show the magnitude of the experimental spectrum, dashed black lines show the total magnitude of the fit, and colored lines show the magnitude of the contribution of individual scattering path contributions; note that these path magnitudes sum coherently and are not directly additive. (d) Schematic of a MAPbX_3_ perovskite structure. The arrows show the first shell Pb-X scattering paths that were considered in the analysis. (e) Deviation of Pb-X bond lengths, Δ*R*, in the mixed-halide perovskites from the corresponding single-halide reference values. The area of each circle is 1/10 of the σ^2^ (MSRD), and error bars reflect discrepancies obtained by fitting under different conditions and k-weights.

For double-halide samples, halide composition is incorporated as a fitting variable in addition to bond distances and mean-squared relative displacements (MSRD) σ^2^; the relative amplitude of each path is constrained to the total expected coordination number of 6, enabling extraction of the average PbX_6_ octahedral composition (Figure 4a,b, paths shown in Figure 4d, *q*-space fits shown in Figures S13–S14). Notably, there is significant uncertainty in the exact values for relative coordination number due to correlations with σ ^2^. The amplitude reduction factor (*S*_0_^2^), whose values for each path are fixed to those from the single-halide fits, also carries over uncertainty. To assess the numerical robustness of the extracted compositions, we repeat fits under variation of data extraction and fitting parameters as minimum *k* values and *k*-weightings (Tables S8–S9). The repeated fits yield an average Br:I ratio of 0.51:0.49 (±0.03), and an average Br:Cl ratio of 0.67:0.33 (±0.02). Note that these EXAFS-derived halide ratios describe local coordination around Pb in the perovskite structure, not the bulk elemental concentration. However, the limited variation across fitting conditions combined with the agreement between XANES, EXAFS, and input chemistry indicate that the film compositions closely match those of the precursor solutions.

Next, we compare the distribution of Pb-X distances with MSRD σ^2^, to validate the fitting protocol and examine trends in materials believed to be miscible.^3,33,34^ In both double-halide materials, both the MSRD and the magnitude of bond-length deviation from the single-halide reference are larger for the longer Pb-X bond. The fitted MSRD σ^2^ are generally larger than that of the single-halide references, which we attribute to static disorder arising additionally from octahedral framework strain, owing to size differences between co-occupying halides on a shared site, assuming random site occupation. Within this framework, the smaller halide is stretched to longer bond distances, whereas the larger halide is compressed below its equilibrium bond length. The bond compression for the larger halide is likely accompanied by angular distortions within the octahedron, which translates to a wider range of bond distances and hence a larger fitted σ^2^. By contrast, the smaller halide is stretched to longer bond lengths with comparatively less angular distortion. The dependence of MSRD σ^2^ on halide size is thus consistent with both halide species mixing within a single-phase shared lattice. The EXAFS-determined Pb-X bond lengths also trend toward intermediate values compared to the single-halide compositions (Figure 4e). For MAPb­(Br_0.6_Cl_0.4_)_3_, the Pb–Cl bond (2.844 Å) is expanded by 0.011 Å, while the Pb–Br bond (2.962 Å) is contracted by 0.016 Å with respect to those in MAPbCl_3_ and MAPbBr_3_, respectively. These changes are consistent with a strained mixed-halide octahedron, where even in a single-phase material, incorporation of halides with varying ionic radius and electronegativity leads us to expect varying Pb-X bond lengths. The single-halide Pb–Cl and Pb–Br bonds have a difference in length of 0.145 Å, which means that the Pb–Cl and Pb–Br bonds each compensate for approximately 7 and 11% of their difference in bond length, respectively, with the Br^–^ bearing the greater strain. A similar, albeit weaker, trend is observed in MAPb­(Br_0.6_I_0.4_)_3_ (1% expansion of the Pb–Br bond and 13% contraction of the Pb–I bond). Across both double-halide compositions, the convergence of Pb-X bond lengths toward intermediate values, together with the size-dependent increase in MSRD, provides consistent signatures of halide mixing, despite not providing conclusive evidence thereof.

After identifying these signatures of double-halide mixing, we likewise probe the nearest-neighbor environment of lead in the single-phase triple-halide perovskite, MAPb­(I_0.2_Br_0.6_Cl_0.2_)_3_ via Pb L_3_-edge EXAFS fitting (Figure 4c). Because we observed two crystallographic phases for nominal MAPb­(I_0.4_Br_0.2_Cl_0.4_)_3_, EXAFS fits assuming a single phase would be misleading, so we only perform quantitative fits for the miscible composition. Like in the double-halide systems, we observe converging Pb-X bond lengths of 2.848 Å, 2.966 Å, and 3.174 Å for X = Cl, Br, and I respectively (Figure 4e). We note that for the triple-halide system, fitting coordination numbers directly is impractical owing to the large number of correlated fit parameters relative to the available data range. Consequently, we hold all path amplitudes *N* fixed according to nominal stoichiometry and, like in the double-halide fits, *S*_0_^2^ is held fixed to the values obtained for single-halide perovskites. Although these compositional assumptions become more complicated for triple-halide perovskites—where the degree of halide mixing is an open question—the consistency between LCA, EXAFS, and input stoichiometry in the double-halide cases provides a validated benchmark for interpreting bond distances and disorder in fully miscible perovskites. Thus, because similar trends in fit results for MAPb­(I_0.2_Br_0.6_Cl_0.2_)_3_ are obtained compared to the double-halide case, it indicates a similar local structure, i.e., halide mixing. The high bromide content of MAPb­(I_0.2_Br_0.6_Cl_0.2_)_3_ is expected to favor miscibility, because the intermediate-radius Br^–^ can readily accommodate both larger I^–^ and smaller Cl^–^ within the octahedral framework. All three Pb-X scattering paths were necessary to effectively reproduce the observed spectra (Table S10), providing further evidence for coordination of all three halides around Pb. To probe potential halide mixing, analysis of best-fit bond distances and disorder is required. In MAPb­(I_0.2_Br_0.6_Cl_0.2_)_3_, as for the double-halides, the longer Pb-X bonds have larger MSRDs than the smaller halides, consistent with halide miscibility in the lattice. Moreover, the Pb-X bond distances converge toward intermediate values (Figure 4e) with shrunken Pb–I and Pb–Br bonds and stretched Pb–Cl bonds. Thus, as for the double-halides, the overall trend is consistent with halide mixing and the changing MSRD is consistent with the proximity of all three halides in a single crystallographic phase.

Because Pb L_3_-edge XAS probes an average over all Pb environments, it cannot distinguish between true mixed-halide coordination (i.e., multiple halide species within individual PbX_6_ octahedra) and nanoscale halide clustering or domain segregation unresolvable via XRD. To address this uncertainty, we employ Br K-edge EXAFS, which directly probes the local environment from the perspective of Br. The Br K-edge spectra (Figure S16) exhibit interference from Pb L_3_-edge oscillations beyond *k* = 10 Å^–1^ (Figure S17), complicating direct quantitative Br EXAFS fits. For a qualitative analysis, we instead apply the Cauchy WT (Figure 5) to resolve distinct scattering contributions in both distance and *k*-space. For MAPbBr_3_ (Figure 5a), the WT reveals a strong intensity feature at R* ≈ 2.5–3 Å, appearing as the overlap of two closely spaced lobes, corresponding to Br ··· Pb scattering paths within the PbBr_6_ octahedron. While there are two inequivalent Br–Pb distances in the low-temperature orthorhombic phase, this peak splitting can be reproduced in FEFF calculations just with a single Br ··· Pb scattering path (Figure S18). This split first shell feature is visible for all compositions measured at the Br K-edge (Figures S19–S22). Heavy backscattering atoms like Pb exhibit nonuniform, oscillatory *k*-dependencies in backscattering amplitude and phase because of relativistic effects.^75^ The observed split can thus be rationalized by interference between the low-*k* and high-*k* amplitudes and phases of a single Br ··· Pb scattering path.

**Figure 5:**
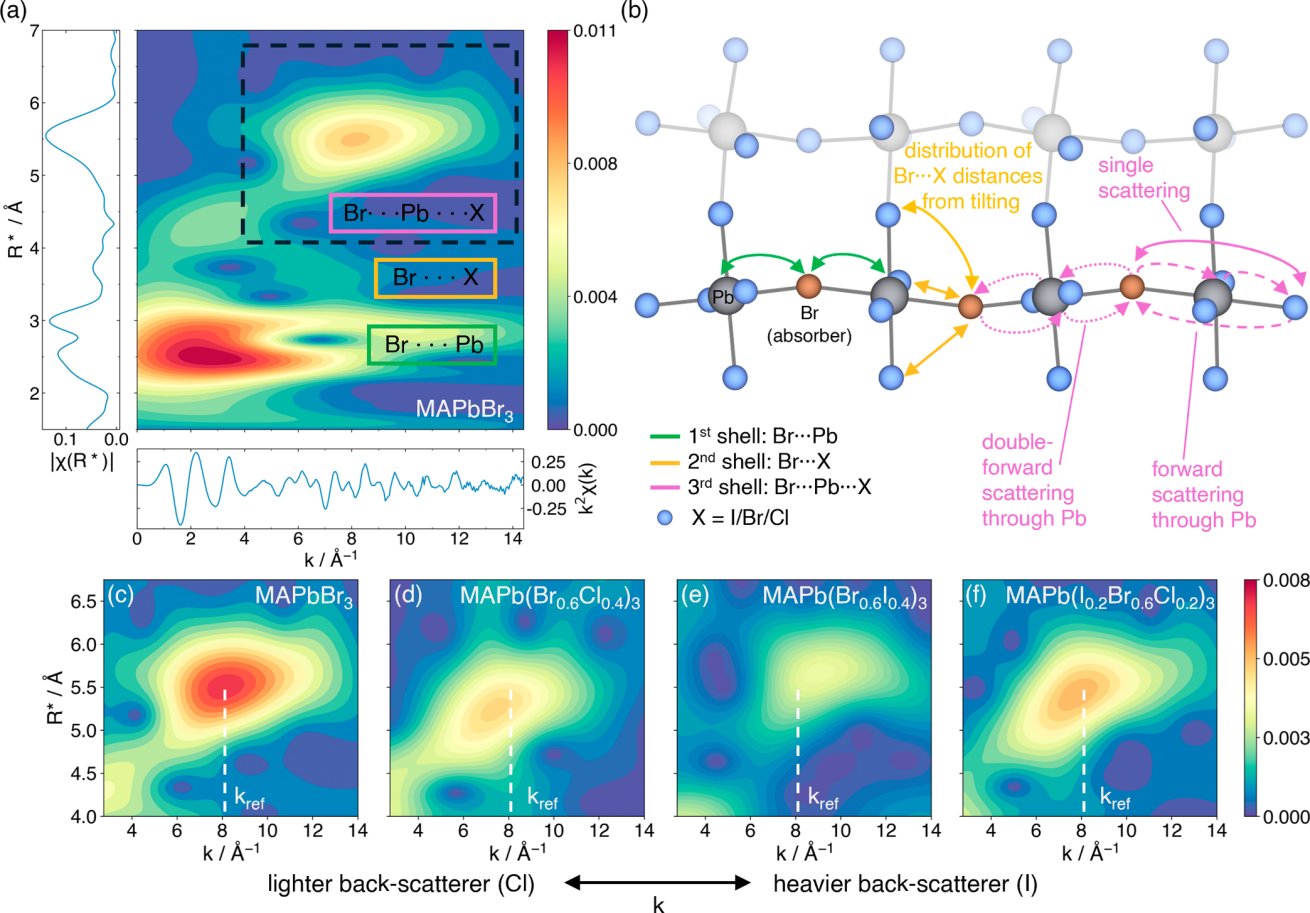
Cauchy wavelet transforms of the Br K-edge EXAFS for (a) MAPbBr_3_, with three contributions labeled: the first, second, and third inorganic shell features. The dashed box indicates the R*-*k* region shown in (c–f). (b) Schematic of representative scattering paths from an absorbing Br within the corner-sharing inorganic perovskite framework indicating the first, second, and distinct third shell scattering paths represented in the WT in (a). WT visualizations of the third shell region, all normalized to the same color scale, are shown for (c) MAPbBr_3_ (d), MAPb­(Br_0.6_Cl_0.4_)_3_ (e), MAPb­(Br_0.6_I_0.4_)_3_ (f) and MAPb­(I_0.2_Br_0.6_Cl_0.2_)_3_. The dashed vertical line marks *k*_ref_, the *k*-position of the Br···Pb···X maximum in pure MAPbBr_3_.

A second, weaker intensity wavelet feature is observed at intermediate R* (∼3.5–5 Å) and distributed widely in *k*, corresponding to the second shell region. In the orthorhombic MAPbBr_3_ lattice, octahedral tilting gives rise to six unique Br···Br single scattering paths that fall within this distance range (Figure 5b). Scattering amplitude calculations for these second shell Br···Br paths (Figure S23,24) show that the superposition of these contributions leads to partial destructive interference, yielding a weak second shell signal. Likewise, in the mixed-halide compositions (full WTs in Figures S19–S22), there is some destructive interference between Br–Br and Br–I scattering paths, as shown by phase shifts close to π above *k* = 8 Å^–1^ (Figure S25). However, the third coordination shell appears in a higher-intensity feature at R* ≈ 5.5 Å and *k* ≈ 8 Å^–1^, corresponding to colinear Br···Pb···Br scattering within a single PbX_6_ octahedron (Figure 5b, pink arrows). At each of the two inequivalent Br sites, three pathways contribute to the third shell: single scattering Br_i_···Br···Br_i_ (Figure 5b, solid pink arrow), forward-scattering Br_i_···Pb···Br···Br_i_ (Figure 5b, dashed pink arrows) and double-forward-scattering Br_i_···Pb···Br···Pb···Br_i_ (Figure 5b, dotted pink arrows). In such nearly colinear geometries, forward scattering through the centric atom (here, Pb) significantly enhances the backscattering strength of the third atom (Br); however, the measured backscattered amplitude can be reduced, owing to out-of-phase destructive interference from the other forward scattering contribution (Figures S23–24).^75,76^ The single scattering Br_i_···Br···Br_i_ contribution remains but is comparatively weaker; the overall intensity is dominated by the amplified forward scattering. For colinear multiple scattering paths, the *k*-dependence of the scattering amplitude remains dominated by the backscattering atom at a scattering angle of 180°, with distinct signatures predicted for each X (Figures S26–S27).^75^ Thus, this third shell EXAFS feature is governed by contributions from halides on the opposite site of the same octahedron, as probed by the absorbing Br. The focusing effect from the forward scattering of the centric Pb amplifies the signal, making it an ideal probe for halide mixing on the single octahedron level. Of note, all contributing scattering paths to this shell have the same effective length and are independent of octahedral tilt in the orthorhombic structure at cryogenic temperatures.

For comparison between single-, double-, and triple-halide samples, the WTs of the third shell are shown in Figure 5b–e, with intensity normalized to that of MAPbBr_3_. Systematic shifts in the R* and *k* positions of the maxima depending on halide composition are apparent: In MAPb­(Br_0.6_Cl_0.4_)_3_ (Figure 5c), this feature is shifted to lower R* and *k* (R* ≈ 5.25 Å^–1^, *k* ≈ 7 Å^–1^), whereas in MAPb­(Br_0.6_I_0.4_)_3_ (Figure 5d) it appears at higher R* and *k* (R* ≈ 5.6 Å^–1^, *k* ≈ 9 Å^–1^) with respect to single halide MAPbBr_3_. These shifts reflect additional scattering from lighter (Cl^–^) and heavier (I^–^) halides opposing the probing Br^–^ in the lead octahedron, respectively. In MAPb­(I_0.2_Br_0.6_Cl_0.2_)_3_ (Figure 5e), the distribution along *k* is broader than in MAPbBr_3_ and the double-halide compositions, evidence of a wider range of backscattering contributions and therefore of halide intermixing on a local scale.

The most prominent difference, however, is not the position but the intensity of the focused third shell feature when comparing the single-halide with the mixed-halide systems. It is greatest in MAPbBr_3_, consistent with a uniform Br–Br neighbor environment, in which all contributions scatter coherently at the same R* and *k*. In the mixed-halide compositions, this intensity is reduced relative to MAPbBr_3_. Note that this reduction in amplitude is not caused by the reduced amount of Br in the structure, as the EXAFS is already normalized to the edge step of the Br K-absorption edge. Instead, it can be rationalized by the introduction of multiple different halide species at the opposite octahedral site: scattering contributions from different halides with significantly different phases lead to partial destructive interference, reducing the net WT intensity. Note that a phase-segregated Br^–^-rich region, where no halide intermixing occurs on a single octahedron level, would be expected to exhibit the same third shell intensity as MAPbBr_3_; the intensity reduction in all mixed-halide compositions thus is a direct consequence of halide mixing at the single octahedron level. The lowest observed WT intensity for MAPb­(Br_0.6_I_0.4_)_3_ can be explained by the calculated scattering phases for X = Br and X = I (Figures S26–27), which differ by approximately π or 3π for the two forward-scattering contributions in the region above *k* = 7 Å^–1^ - both antiphase - leading to strong destructive interference when both backscatterers are present at similar *R*. In MAPb­(Br_0.6_Cl_0.4_)_3_, there is likewise destructive interference between the double forward scattering paths for X = Br and X = Cl due to a phase difference of approximately 3π above *k* = 7 Å^–1^, but there is no such trend for the single forward scattering paths, leading to somewhat higher observed intensity. Meanwhile, in triple-halide MAPb­(I_0.2_Br_0.6_Cl_0.2_)_3_ the third shell WT intensity is intermediate between the double-halides and MAPbBr_3_. This reflects the constructive interference between I and Cl double forward scattering contributions, which have the same phase at *k* ∼ 7.5 Å^–1^ and similar phase at greater *k* (Figure S27). Thus, based both on the distribution of the third coordination shell in *k* and R* and the moderately reduced intensity, we can conclude that bromide is intermixed both with iodide and with chloride on a single octahedron level in the triple-halide perovskite MAPb­(I_0.2_Br_0.6_Cl_0.2_)_3._ We note that the Br K-edge analysis directly probes only the halides neighboring Br and therefore cannot, by itself, establish Cl–I mixing within octahedra that contain no Br; a future I K-edge study could provide such direct evidence. However, knowing that Br and I intermix on the single octahedron level, coupled with results obtained at the Pb L_3_-edge showing the direct impact of Cl on the Pb–I bond distance (see Figure 4e), strongly suggests all three halides in local proximity.

## Conclusions

We have demonstrated the formation of a solution-processed triple-halide perovskite thin-film in which all three halides coordinate within a single phase, with halide mixing evident down to the scale of individual PbX_6_ octahedra. Using XRD, we establish that Br^–^ content is a key determinant of phase stability in triple-halide compositions: a nominal 60% Br^–^ composition yields a highly oriented and single-phase film, MAPb­(I_0.2_Br_0.6_Cl_0.2_)_3_, whereas a lower Br^–^ content (20%) results in phase segregation into distinct I^–^-rich and Cl^–^-rich domains. We perform linear combination analysis of the Pb L_3_-edge XANES, using experimental spectra for single-halide MAPbX_3_ as standards, which demonstrates that in mixed compositions, all halides coordinate in a perovskite structure in ratios aligned with solution stoichiometry. Wavelet transforms (WTs) of the Pb L_3_ EXAFS show that in the single-phase triple-halide MAPb­(I_0.2_Br_0.6_Cl_0.2_)_3_, more Pb-X bonds take on intermediate lengths compared to the phase-segregated composition, providing a local analogue to the trends observed in bulk-averaged XRD. Quantitative EXAFS fitting establishes these consistent signatures of halide mixing: for double-halides MAPb­(Br_0.6_Cl_0.4_)_3_ and MAPb­(Br_0.6_I_0.4_)_3_ and triple-halide MAPb­(I_0.2_Br_0.6_Cl_0.2_)_3_, we observe convergence of Pb-X bond lengths toward intermediate values relative to single-halide references, and systematically increased disorder (MSRD), consistent with strain from accommodating mixed ionic radii. Critically, Br K-edge WTs demonstrate third shell halide-to-halide backscattering, amplified by forward scattering through the centric Pb atom, forming a signature that is highly sensitive to the halides on the same octahedron as the absorbing Br atom. For the double-halide compositions MAPb­(Br_0.6_Cl_0.4_)_3_ and MAPb­(Br_0.6_I_0.4_)_3_, systematic shifts in the third shell WT intensity directly demonstrate that Br occupies the same PbX_6_ octahedron with Cl or I, respectively. In triple-halide MAPb­(I_0.2_Br_0.6_Cl_0.2_)_3_, the *k*-dependence of this feature reveals a distribution of halide species at the octahedral sites opposite Br, providing evidence that halide mixing with Br^–^ occurs within individual octahedra in this single-phase triple-halide perovskite. These results connect average crystallographic structure, Pb-centered coordination, and Br-centered local environment to establish mixed-halide incorporation from the bulk crystal structure down to the individual PbX_6_ octahedron. These results highlight the remarkable structural flexibility of mixed-halide perovskites and provide a robust spectroscopic framework for characterizing complex halide incorporation. As the compositional space of mixed halide perovskites continues to be explored, understanding and controlling halide coordination will be essential for the rational design of stable, high-performance wide-bandgap absorbers.

## Supplementary Material



## Notes

Normalization and background subtraction of XAS data, quantitative fits of single-halide Pb EXAFS, Pb EXAFS fit values and plots in q for mixed-halide perovskites, full wavelet transform images, XRD on polyimide substrates, structural parameters from XRD fits, and ab initio amplitudes and phases of different EXAFS scattering paths (PDF)

## ⊥

Department of Chemistry, University of Toronto, Toronto, Ontario M5S 3H6, Canada
