# Supplementary material for: Probing Local Coordination and Halide Miscibility in Single‑, Double‑, and Triple-Halide Perovskites Using EXAFS
Source: J Phys Chem C Nanomater Interfaces. 2026 Aug 5;130(32):11446–57. doi: 10.1021/acs.jpcc.6c03620 (PMC13479783; doi:10.1021/acs.jpcc.6c03620)
Supplement: Supplementary file 1 [file jp6c03620_si_001.pdf]

Supporting Information: Probing local coordination and halide miscibility in single-, double-, and triple-halide perovskites using EXAFS

Sonia S. Mulgund,[1†] Esther Y.-H. Hung,[2] Leslie Bostwick,[3] Ashley Galbraith,[1] Owen M. Romberg,[4] Justus Just,[2] Rebecca A. Belisle[1*]*

[1] Department of Physics and Astronomy, Wellesley College, Wellesley, Massachusetts 02481, United States

[2] MAX IV Laboratory, Lund University, 22484 Lund, Sweden

[3] Olin College of Engineering, Needham, Massachusetts 02492, United States

[4] Department of Chemistry, Wellesley College, Wellesley, Massachusetts 02471 United States

X-ray Diffraction Analysis

All X-ray diffraction images were collected at grazing incidence of 3° and approximate detector distance of 175 mm. Image calibration was done using a LaB_6 standard in pyFAI,[1] followed by grazing incidence geometry correction using pygix.[2] Images and diffraction patterns represent the average diffraction from three subsequent scans normalized for beam intensity. Full azimuthal integrations (chi width of 170°) were completed for all samples. Relevant structural parameters (e.g. FWHM of diffracted peaks, peak area, etc.) were determined by curve fitting background subtracted integrated data to a pseudo-Voight peak model using a non-linear least squares method. Lattice parameters were determined from Pawley fit performed using GSAS-II over the Q≈1.0–3.3 $Å^{-1}$ data range for all apparent mixed compositions.[3] Lattice parameters and other structural parameters are presented in **Table S1**. Data and fits from Pawley fit assuming $Pm\bar{3}m$ symmetry shown in **Figures S32-34.**

EXAFS Analysis

Br K-edge EXAFS data were processed and Cauchy wavelet transforms W(*k*,*R**) computed using a background removal parameter of r_{bkg} ~1.6 Å, a *k*-range of 2.5-14.45 $Å^{-1}$ with k^2 weighting. For a low r_{bkg} (~1.0 Å), a low intensity feature which is broad in *k* appears at low *R** in all Br *k*-edge WTs (**Figure S30**). This is attributed to residual background rather than a structural coordination shell. The higher r_{bkg} value suppresses this without affecting the first shell Br-Pb feature. FEFF6 (implemented in Artemis) was used to calculate scattering paths for the quantitative Pb L_3-edge fits and FEFF8 (implemented in Larch) was used to calculate scattering amplitudes and phases as shown in **Figure S10** and **Figures S25-S27**.

Chemistry	(100) Pseudocubic lattice parameter (Å)		(100) Pseudo cubic FWHM ($Å^{-1}$)	
$MAPb(Br_{0.6}I_{0.4})_3$	6.04*		0.0162	
$MAPb(Br_{0.6}Cl_{0.4})_3$	5.81*		0.0135	
$MAPb(Br_{0.6}I_{0.2}Cl_{0.2})_3$	5.91*		0.0160	
$MAPb(Br_{0.2}I_{0.4}Cl_{0.4})_3$	6.16	5.76	0.0167	0.0170

Table S1. *Cubic lattice parameter determined from Pawley fit. All other Structural parameters from pseudo-Voigt peak fitting of diffraction data.

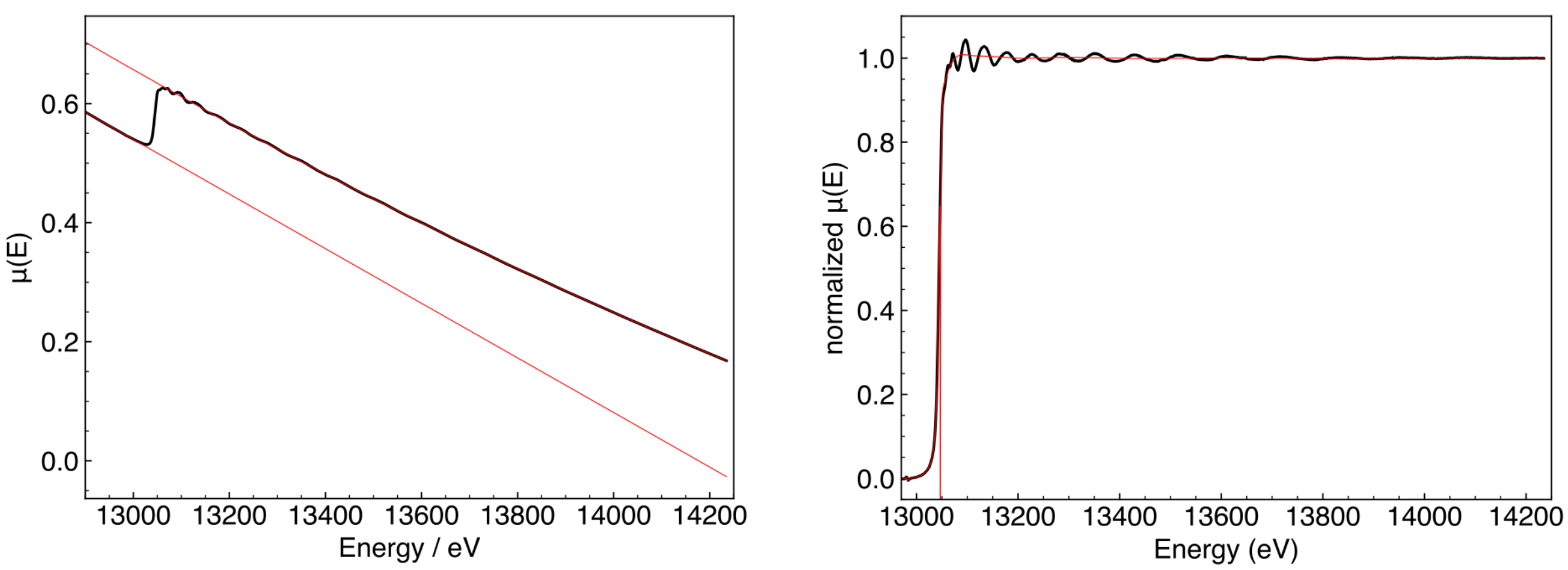

Figure S1. Pre-and post-edge polynomials and normalization of Pb L3 EXAFS for $MAPbI_3$. Data in black.

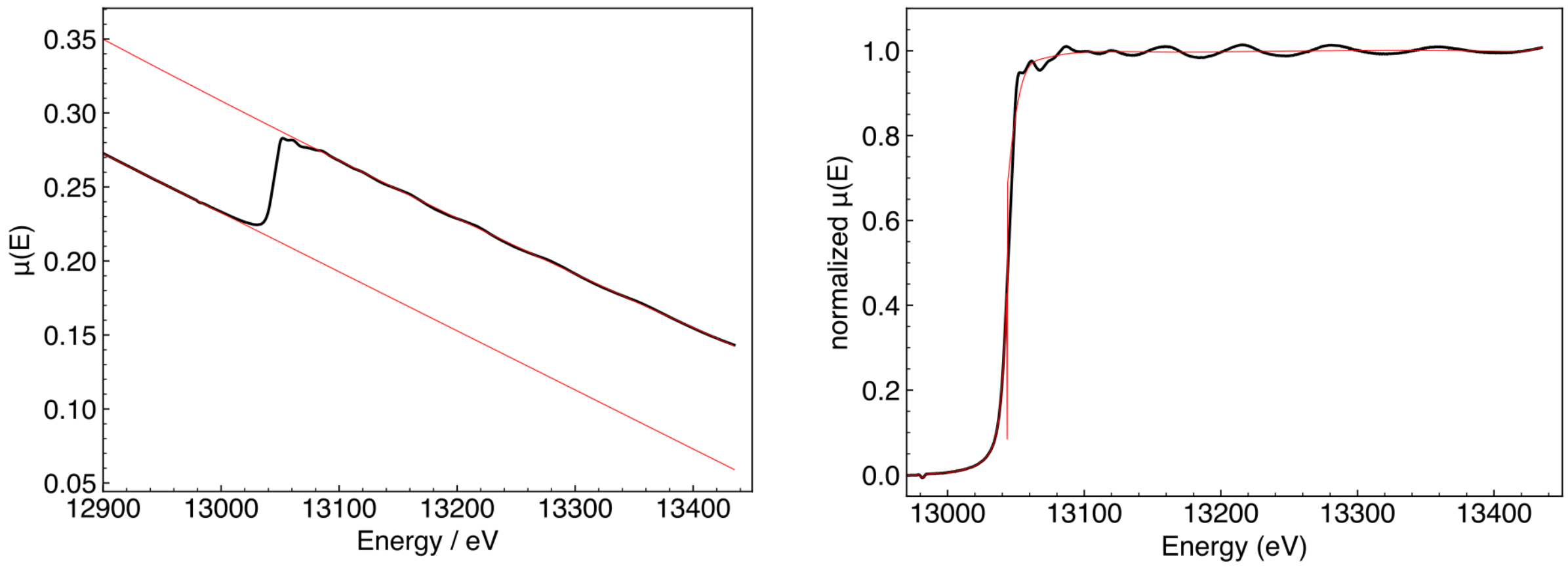

Figure S2. Pre-and post-edge polynomials and normalization of Pb L3 EXAFS for $MAPbBr_3$. Data in black.

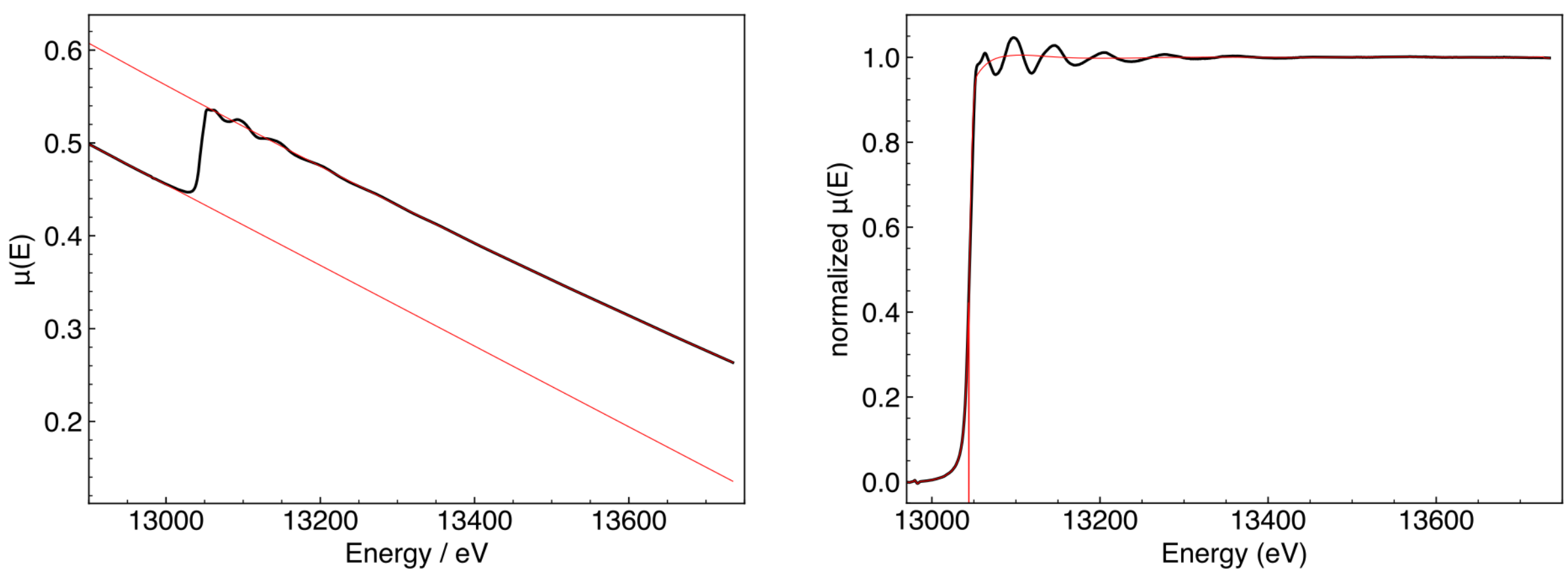

Figure S3. Pre-and post-edge polynomials and normalization of Pb L3 EXAFS for $MAPbCl_3$. Data in black.

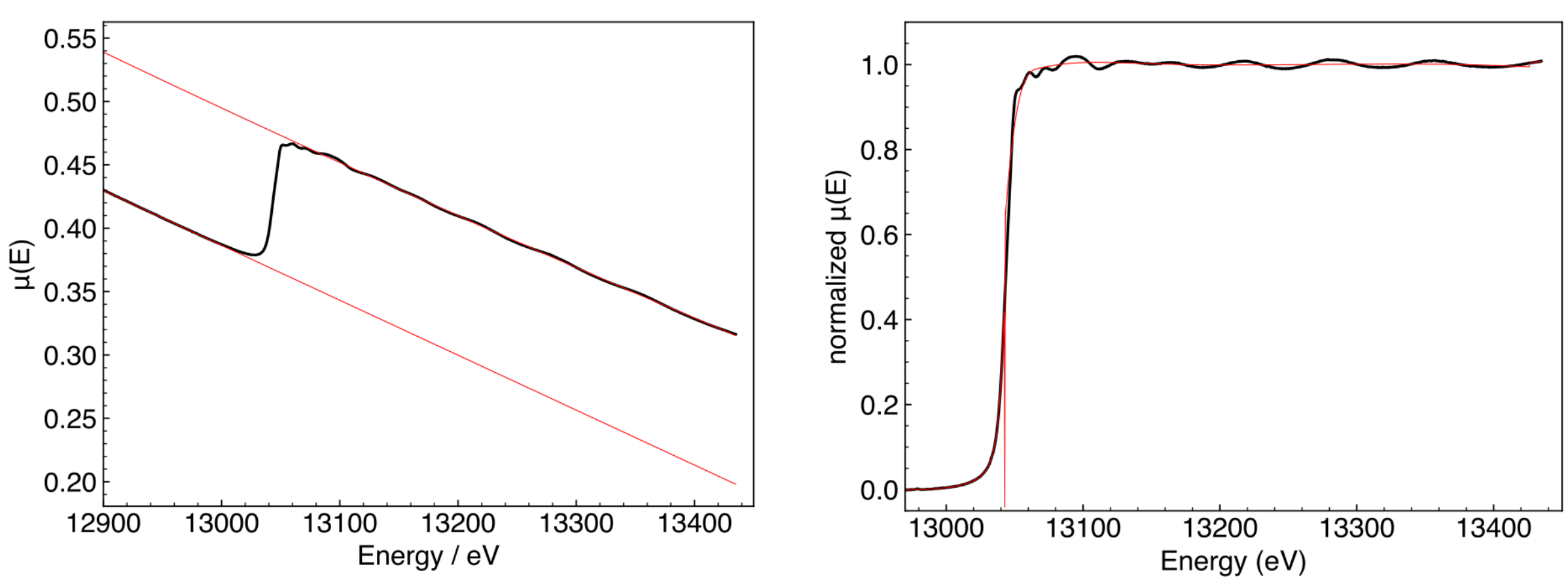

Figure S4. Pre-and post-edge polynomials and normalization of Pb L3 EXAFS for $MAPb(Br_{0.6}I_{0.4})_3$. Data in black.

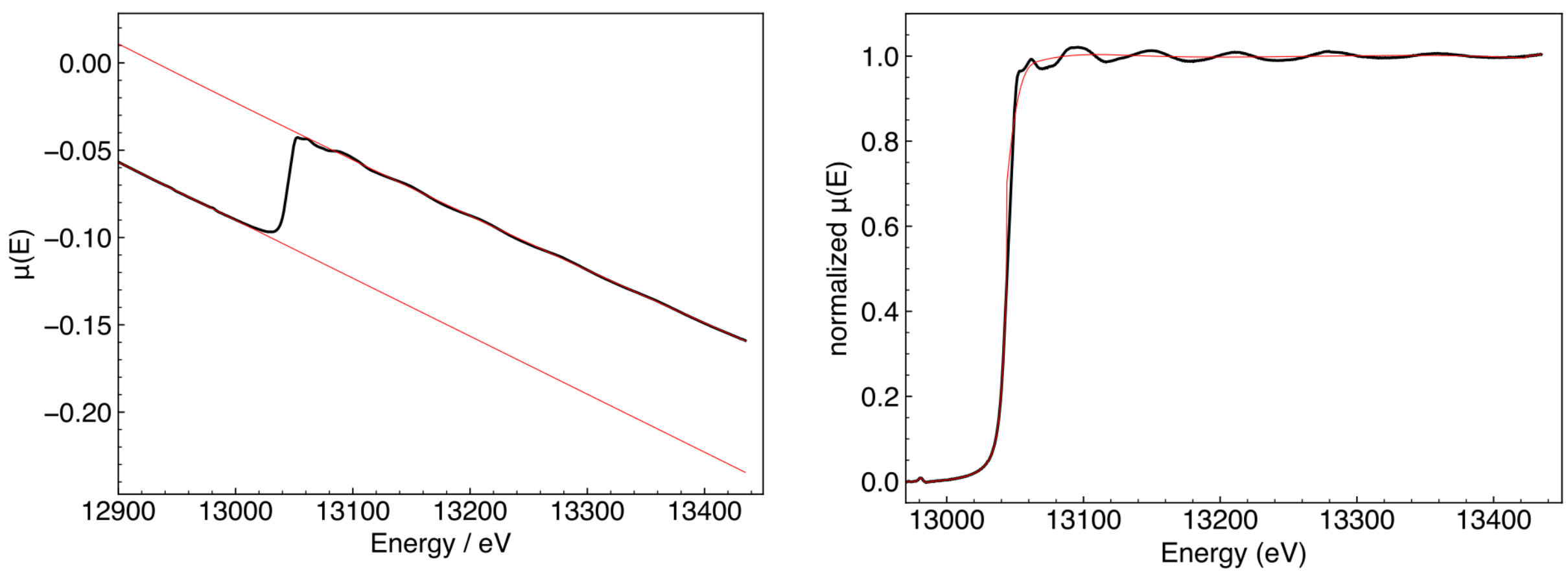

Figure S5. Pre-and post-edge polynomials and normalization of Pb L3 EXAFS for $MAPb(Br_{0.6}Cl_{0.4})_3$. Data in black.

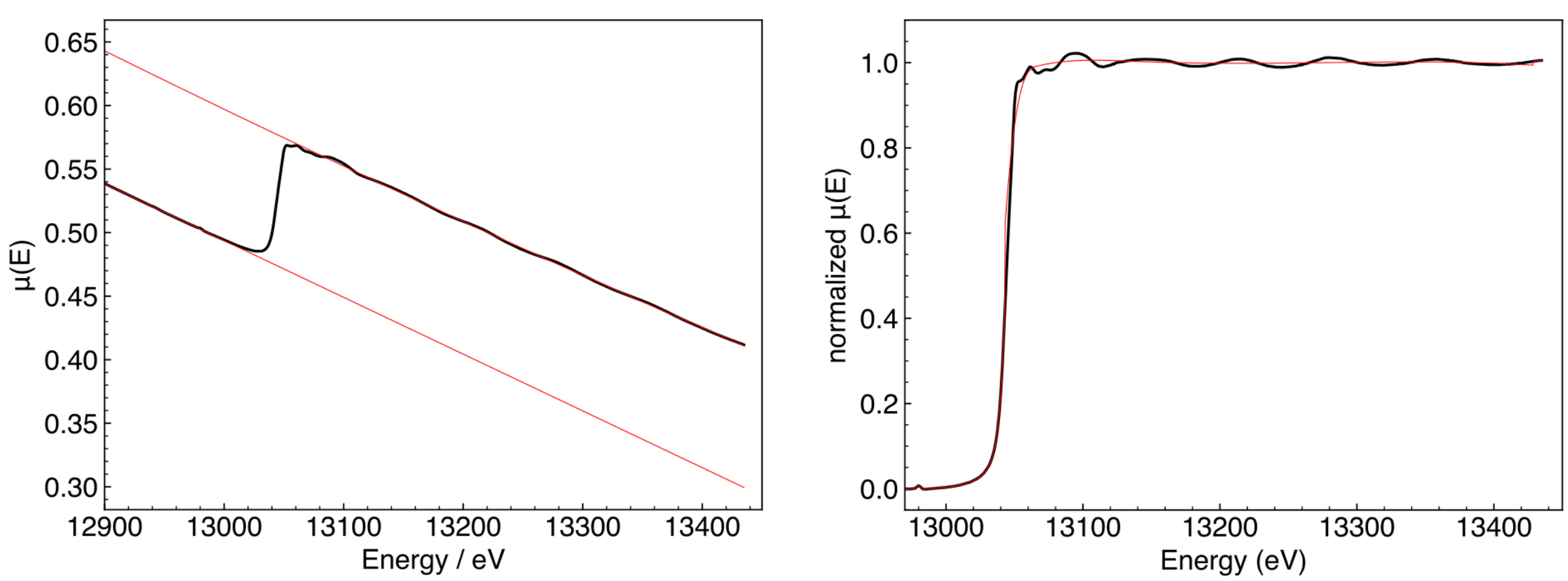

Figure S6. Pre-and post-edge polynomials and normalization of Pb L3 EXAFS for $MAPb(I_{0.2}Br_{0.6}Cl_{0.2})_3$. Data in black.

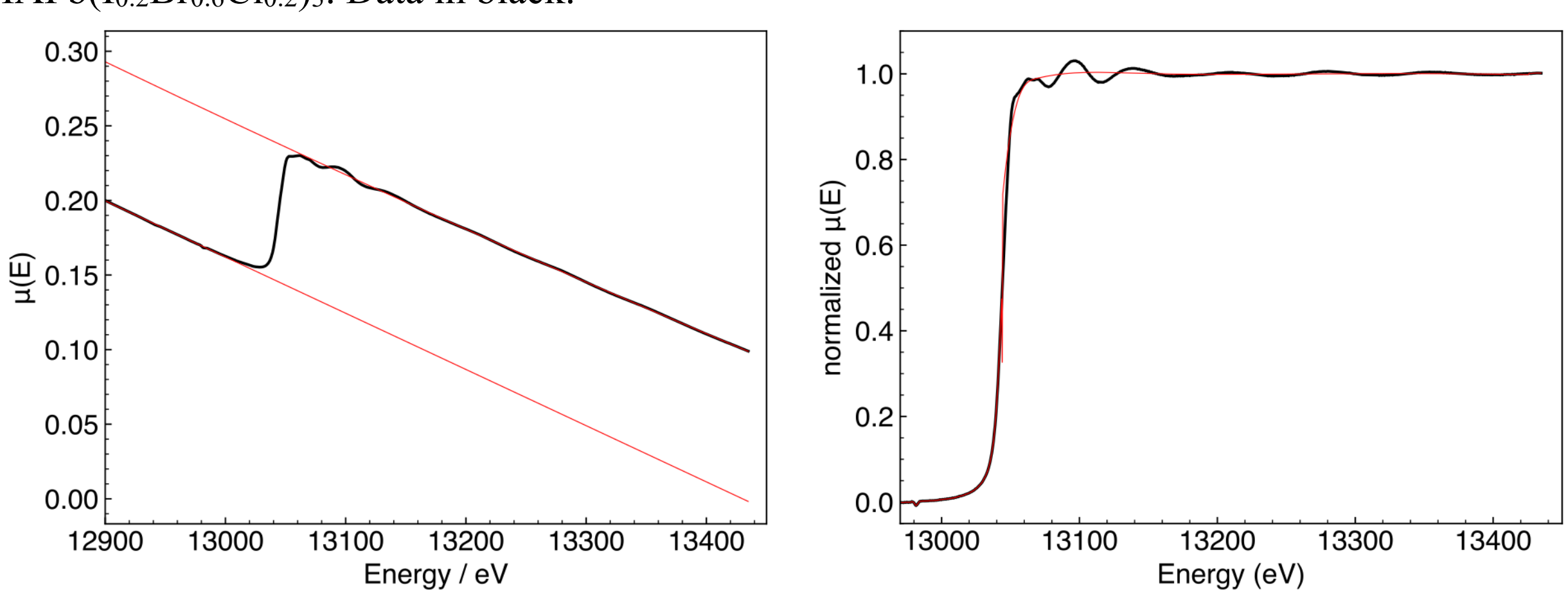

Figure S7. Pre-and post-edge polynomials and normalization of Pb L3 EXAFS for $MAPb(I_{0.4}Br_{0.2}Cl_{0.4})_3$. Data in black.

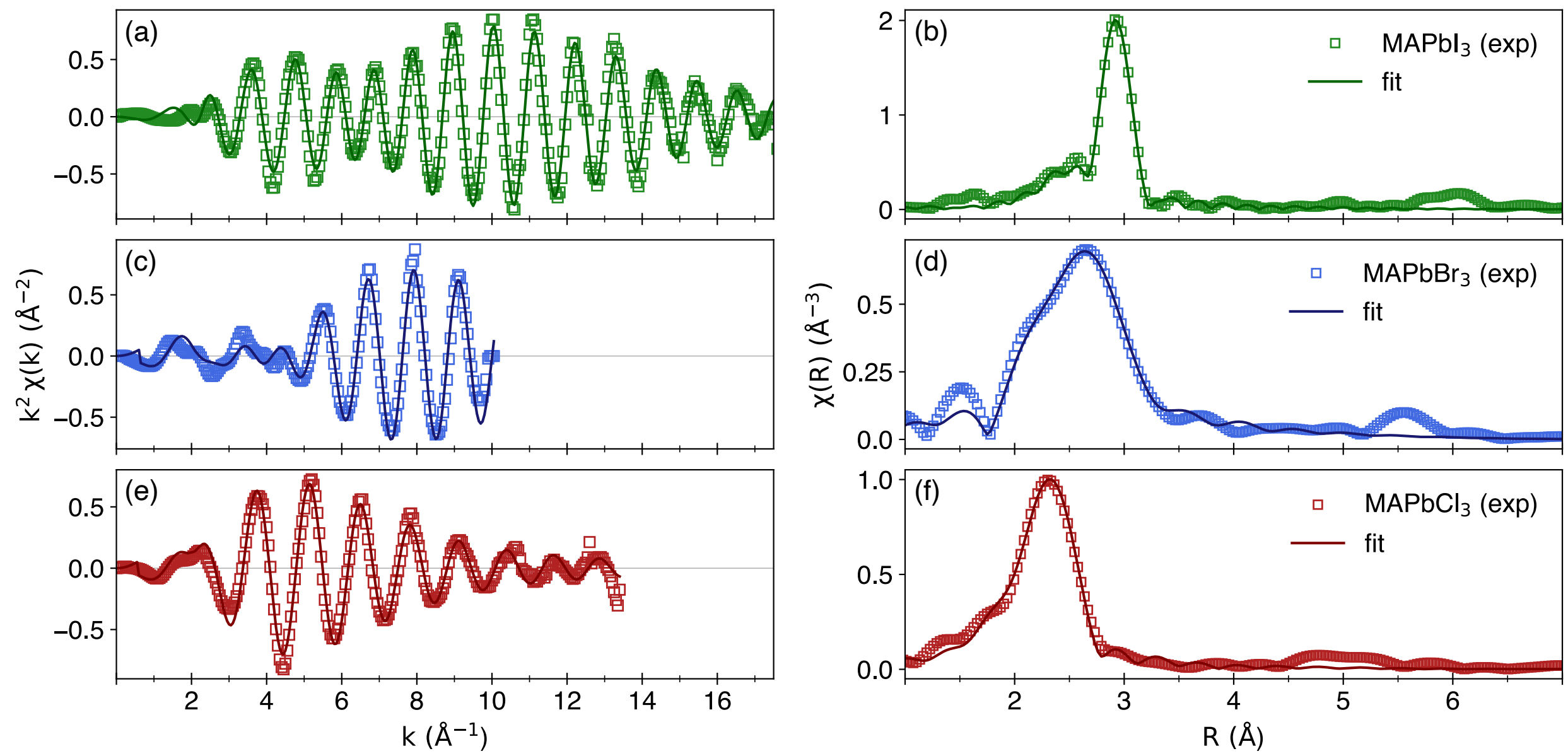

Figure S8. Pb L_3 EXAFS data with first shell fits for single-halide samples. (b), (d), and (f) show the Fourier transform in R, while (a), (c), and (e) show the q-space fit overlaid on the $\chi(k)$ data. (a-b): $MAPbI_3$. (c-d): $MAPbBr_3$. (e-f): $MAPbCl_3$.

Material	Authors	Pb-X distance	Temperature
$MAPbI_3$	Weller et al.[4]	3.19 Å	100 K
$MAPbBr_3$	Swainson et al.[5]	3.04 Å	11 K
$MAPbCl_3$	Chi et al.[6]	2.73-3.02 Å	80 K

Table S2. Information from CIFs for single-halide perovskites (all *Pnma*) used as inputs to ATOMS. $MAPbCl_3$ has a lower symmetry PbX_6 octahedron, resulting in a spread of Pb-X bond distances.

Sample	k-weight	R-range (Å)	ΔE_0 (eV)	S_0^2	$R_{Pb\text{-}X}$ (Å)	σ^2 (Å^2)
$MAPbI_3$	3	2-3.3	-0.71	0.85	3.181	0.004
$MAPbBr_3$	3	1.75-3.4	1.51	0.96	2.978	0.0071
$MAPbCl_3$	2,3	1.35-3	1.30	1.09	2.723-3.017	0.0045

Table S3. First-shell EXAFS fits (performed in back-Fourier transformed q-space) from each single-halide perovskite composition, as shown in **Figure S1**. The reduced symmetry in the low-temperature orthorhombic phase of $MAPbCl_3$ results in multiple Pb-Cl bond distances, which were all constrained to change by the same fractional amount.

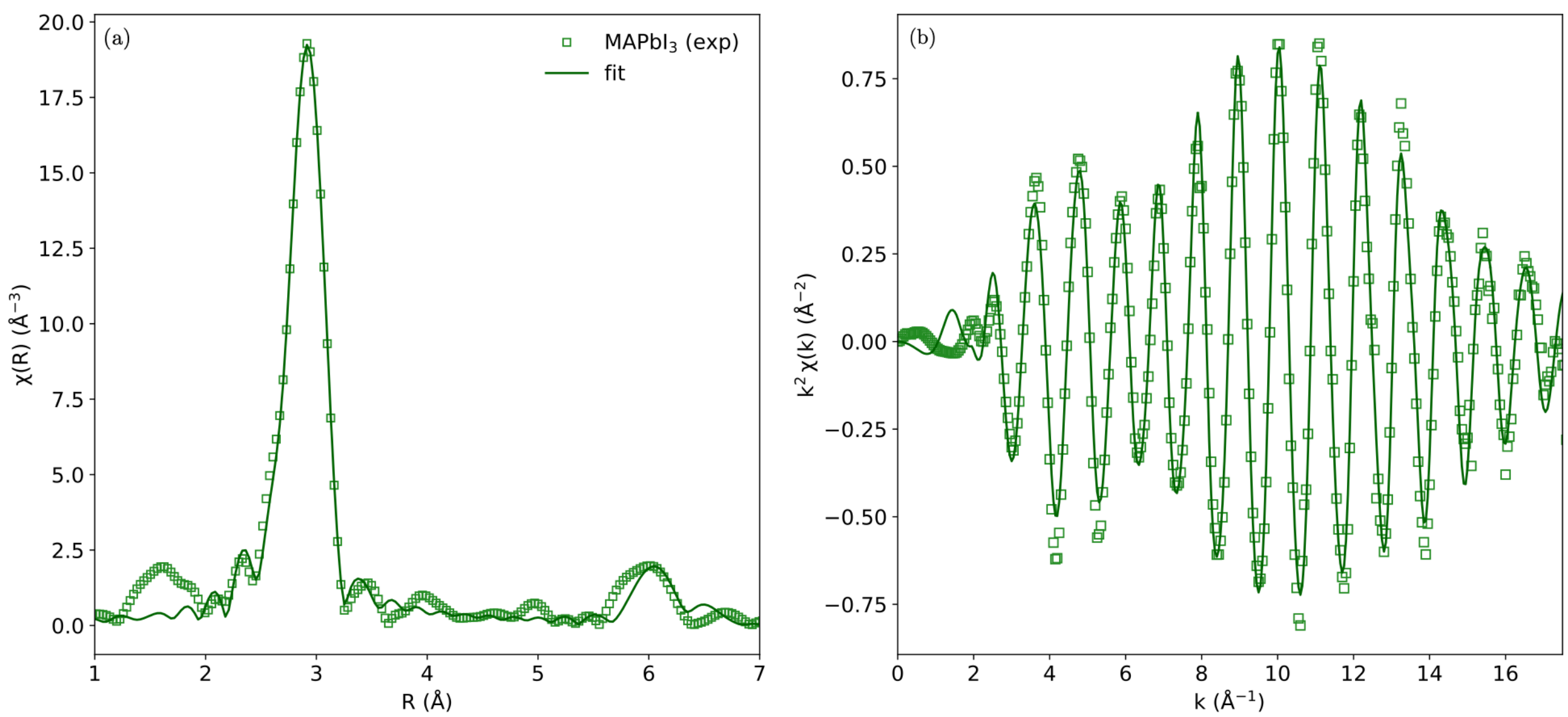

Figure S9. Pb L3 EXAFS data for $MAPbI_3$ with second shell fit, performed in back-Fourier transformed q-space. (a) Fit to $\chi(R)$. (b) Fit to $\chi(k)$. All paths generated by ATOMS containing Pb and I atoms with an effective path length less than 6.5 Å were incorporated into the fit. Values for S_0^2 and ΔE_0 were fixed to the first-shell values (**Table S2**). The first shell Pb-I-Pb scattering path was fit to one set of values for ΔR and σ^2, and all other paths were fit to a second set of values for ΔR and σ^2. For the first shell R = 3.181 Å and σ^2 = 0.0041 Å^2, and for the second shell ΔR = 0.018 Å and σ^2 = 0.0048 Å^2.

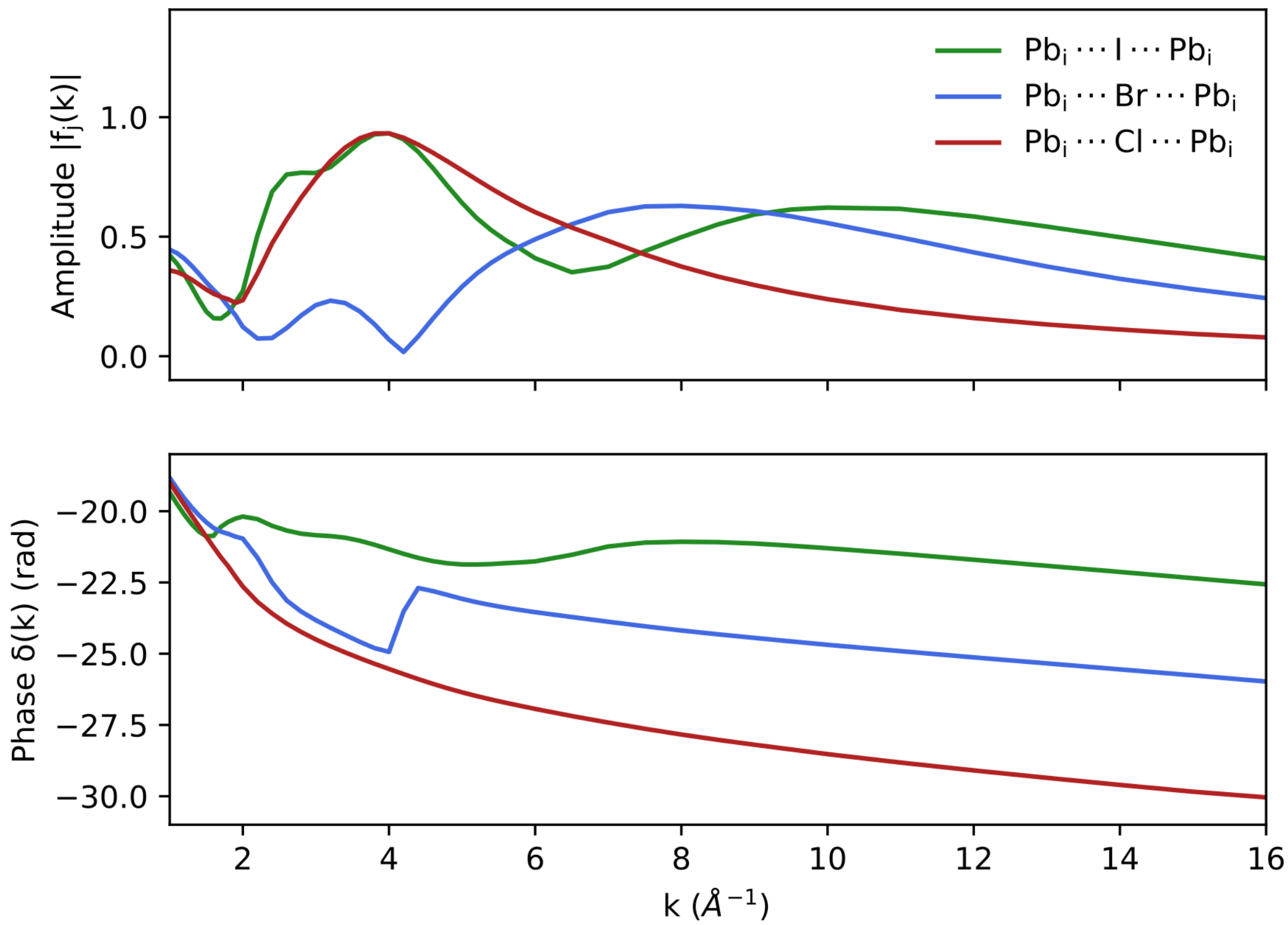

Figure S10. Calculated scattering amplitudes and phase shifts for Pb-X single scattering paths as calculated in FEFF from the structures in **Table S1** using the σ^2 from **Tables S4-S6**.

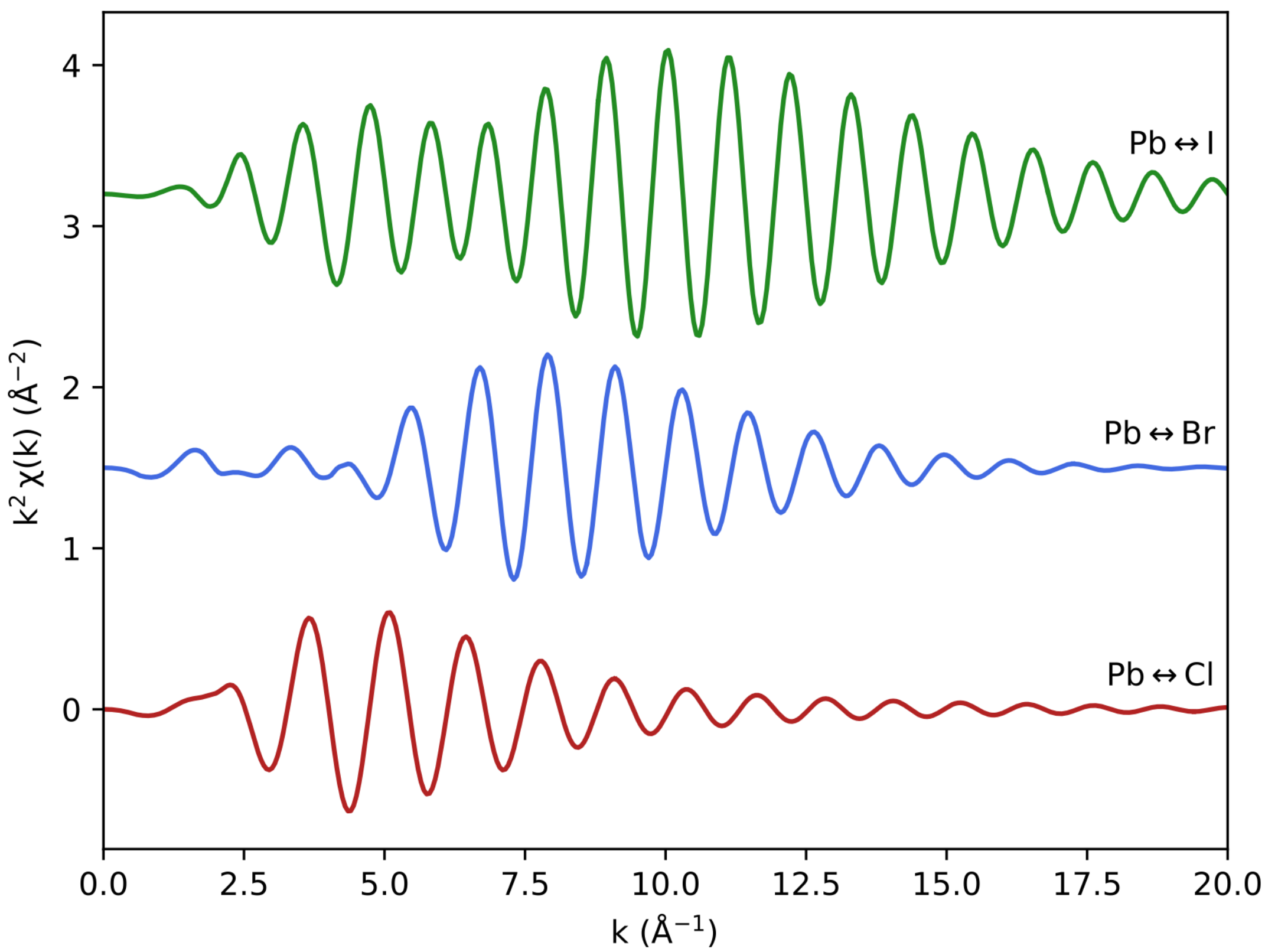

Figure S11. Calculated χ(k) for the three single-halide scattering paths using the fit parameters described in **Tables S4-S6**.

Sample	k-weight	R-range (Å)	ΔE_0 (eV)	σ^2_{Cl} (Å^2)	σ^2_{Br} (Å^2)	σ^2_{I} (Å^2)
$MAPb(Br_{0.6}Cl_{0.4})_3$	3	1.75-3.4	0.46	0.0068	0.0098	---
$MAPb(Br_{0.6}I_{0.4})_3$	3	1.8-3.4	1.12	---	0.0047	0.0093
$MAPb(I_{0.2}Br_{0.6}Cl_{0.2})_3$	2,3	1.85-3.5	2.63	0.0058	0.0068	0.0086
Sample	R_{Pb-Cl}(Å)	R_{Pb-Br}(Å)	R_{Pb-I}(Å)	n_{Cl}	n_{Br}	n_{I}
$MAPb(Br_{0.6}Cl_{0.4})_3$	2.846	2.963	---	2.07	3.93	---
$MAPb(Br_{0.6}I_{0.4})_3$	---	2.979	3.156	---	2.93	3.07
$MAPb(I_{0.2}Br_{0.6}Cl_{0.2})_3$	2.869	2.974	3.175	1.2	3.6	1.2

Table S4. First-shell EXAFS fits (performed in back-Fourier transformed q-space) from each mixed-halide perovskite composition, as shown in **Figure 4a-d**.

fit	amp	enot	del_r	ss
kw = 3				
kmin = 1.5	0.847203	-0.77373	-0.00918	0.004078
kmin = 1.75	0.8472	-0.77099	-0.00917	0.004078
kmin = 2	0.847224	-0.76649	-0.00916	0.004078
2.25	0.847368	-0.76189	-0.00914	0.004079
2.5	0.847564	-0.75828	-0.00913	0.00408
kw = 2,3				
1.5	0.867005	-0.67038	-0.00873	0.004158
1.75	0.867641	-0.6542	-0.00867	0.004161
2	0.86837	-0.63325	-0.0086	0.004164
2.25	0.870031	-0.61761	-0.00854	0.004171
2.5	0.872055	-0.60376	-0.00849	0.004179
AVGS	0.858166	-0.70106	-0.00888	0.004123
STDS	0.011522	0.071174	0.000298	4.66E-05

Table S5. First-shell EXAFS fits (performed in back-Fourier transformed q-space) for $MAPbI_3$ for a range of k-weights and minimum k-values, which generated the error bar for this composition in **Figure 4e**.

fit	amp	enot	del_r	ss
kw = 3				
kmin = 1.5	0.955872	1.506708	-0.06277	0.007097
kmin = 1.75	0.956511	1.510931	-0.06274	0.007103
kmin = 2	0.955914	1.505223	-0.06277	0.007097
2.25	0.95646	1.50862	-0.06275	0.007102
2.5	0.959319	1.541919	-0.06252	0.00713
kw = 2,3				
1.5	0.957248	1.725591	-0.06141	0.007106
1.75	0.963817	1.764445	-0.06111	0.007164
2	0.960667	1.696386	-0.06153	0.007134
2.25	0.958456	1.653289	-0.06181	0.007114
2.5	0.962555	1.726037	-0.06133	0.007155
AVGS	0.958682	1.613915	-0.06208	0.00712
STDS	0.002849	0.108647	0.000694	2.45E-05

Table S6. First-shell EXAFS fits (performed in back-Fourier transformed q-space) for $MAPbBr_3$ for a range of k-weights and minimum k-values, which generated the error bar for this composition in **Figure 4e**.

fit	amp	enot	frac	ss	delr_1	delr_2	delr_3	delr_4
kw = 3								
kmin = 1.5	1.118937	0.901241	0.996622	0.00473	-0.00922	-0.00958	-0.00969	-0.01022
kmin = 1.75	1.118273	0.895801	0.996606	0.004725	-0.00926	-0.00962	-0.00974	-0.01026
kmin = 2	1.11875	0.886771	0.996586	0.004728	-0.00932	-0.00968	-0.0098	-0.01033
2.25	1.121972	0.888445	0.996595	0.004751	-0.00929	-0.00965	-0.00977	-0.0103
2.5	1.127111	0.902246	0.996635	0.004788	-0.00918	-0.00954	-0.00965	-0.01017
kw = 2,3								
1.5	1.072634	1.277381	0.997659	0.004361	-0.00639	-0.00664	-0.00672	-0.00708
1.75	1.069779	1.265478	0.997614	0.004336	-0.00651	-0.00676	-0.00684	-0.00721
2	1.069648	1.242589	0.997546	0.004334	-0.0067	-0.00696	-0.00704	-0.00742
2.25	1.077024	1.2453	0.997562	0.004396	-0.00665	-0.00691	-0.00699	-0.00737
2.5	1.089191	1.301203	0.997729	0.004498	-0.0062	-0.00644	-0.00652	-0.00687
AVGS	1.098332	1.080645	0.997115	0.004565	-0.00787	-0.00818	-0.00828	-0.00872
STDS	0.024636	0.196514	0.000536	0.000196	0.001464	0.001521	0.001539	0.001622

Table S7. First-shell EXAFS fits (performed in back-Fourier transformed q-space) for $MAPbCl_3$ for a range of k-weights and minimum k-values, which generated the error bar for this composition in **Figure 4e**.

fit	enot	delr_br	delr_i	ss_br	ss_i	n_i	n_br
kw = 3							
kmin = 1.5	1.179582	-0.06099	-0.03394	0.004803	0.009245	3.045457	2.954543
kmin = 1.75	1.163692	-0.06111	-0.03395	0.004782	0.009275	3.052434	2.947566
kmin = 2	1.118133	-0.0614	-0.03411	0.004738	0.00933	3.068721	2.931279
2.25	1.038902	-0.06198	-0.0343	0.004622	0.009491	3.107443	2.892557
2.5	1.032948	-0.06257	-0.03349	0.004335	0.009963	3.189856	2.810144
kw = 2,3							
1.5	1.278106	-0.05827	-0.03552	0.00588	0.007344	2.742385	3.257615
1.75	1.309714	-0.05821	-0.03519	0.00583	0.007442	2.754837	3.245163
2	1.236263	-0.05869	-0.03556	0.005721	0.007602	2.791512	3.208488
2.25	1.036507	-0.06005	-0.03651	0.005446	0.007995	2.882759	3.117241
2.5	0.946705	-0.06163	-0.03594	0.00483	0.008985	3.061896	2.938104
AVGS	1.134055	-0.06049	-0.03485	0.005099	0.008667	2.96973	3.03027
STDS	0.119792	0.001593	0.001018	0.000563	0.000968	0.161754	0.161754

Table S8. First-shell EXAFS fits (performed in back-Fourier transformed q-space) for $MAPb(Br_{0.6}I_{0.4})_3$ for a range of k-weights and minimum k-values, which generated the error bar for this composition in **Figure 4e**.

fit	enot	delr_br	delr_cl	ss_br	ss_cl	n_cl	n_br
kw = 3							
kmin = 1.5	0.515593	-0.07738	0.004276	0.009828	0.006678	2.061879	3.938121
kmin = 1.75	0.456591	-0.07741	0.003964	0.009779	0.006788	2.068427	3.931573
kmin = 2	0.376799	-0.0774	0.003592	0.009686	0.006994	2.084304	3.915696
2.25	0.372137	-0.07719	0.003717	0.00959	0.007194	2.109091	3.890909
2.5	0.519706	-0.07682	0.004645	0.009604	0.007143	2.121358	3.878642
kw = 2,3							
1.5	0.644961	-0.08009	0.002129	0.010985	0.004487	1.837344	4.162656
1.75	0.548625	-0.08011	0.001549	0.010938	0.004587	1.838473	4.161527
2	0.415875	-0.08011	0.000796	0.010843	0.004784	1.84773	4.15227
2.25	0.407914	-0.07969	0.001179	0.010661	0.005122	1.884738	4.115262
2.5	0.624267	-0.07888	0.003019	0.010538	0.005328	1.928566	4.071434
AVGS	0.488247	-0.07851	0.002887	0.010245	0.005911	1.978191	4.021809
STDS	0.098341	0.001393	0.001376	0.000595	0.001141	0.120928	0.120928

Table S9. First-shell EXAFS fits (performed in back-Fourier transformed q-space) for $MAPb(Br_{0.6}Cl_{0.4})_3$ for a range of k-weights and minimum k-values, which generated the error bar for this composition in **Figure 4e**.

fit	enot	delr_cl	delr_br	delr_i	ss_cl	ss_br	ss_i
kw = 3							
kmin = 1.5	1.4459168	0.00232891	-0.07673137	-0.01258876	0.00372107	0.00856729	0.00513212
kmin = 1.75	1.41373272	0.00202601	-0.07684082	-0.01280089	0.00373347	0.00857563	0.00511026
kmin = 2	1.28172063	0.00024242	-0.07733305	-0.01367189	0.00372018	0.00867957	0.00491284
2.25	1.06427275	-0.00318504	-0.07843807	-0.01478913	0.00356897	0.00893906	0.00451393
2.5	1.01443497	-0.00477677	-0.0792632	-0.01449042	0.00331344	0.00914684	0.00430529
kw = 2,3							
1.5	2.08502917	0.01788753	-0.06964993	-0.01681804	0.00557795	0.00727148	0.00714118
1.75	2.31410011	0.02242141	-0.06870423	-0.01602519	0.00568153	0.00703214	0.00786916
2	1.98080244	0.01729762	-0.06950865	-0.01866363	0.00583674	0.00723601	0.00712295
2.25	1.42811921	0.00679478	-0.07166264	-0.02084915	0.0055702	0.0078003	0.00573637
2.5	1.25759033	0.00127307	-0.0733836	-0.01959332	0.00489999	0.00828811	0.00502115
AVGS	1.52857191	0.00623099	-0.07415156	-0.01602904	0.00456235	0.00815364	0.00568653
STDS	0.44417207	0.00956833	0.00403859	0.00289095	0.00103754	0.00076377	0.00124317

Table S10. First-shell EXAFS fits (performed in back-Fourier transformed q-space) for $MAPb(I_{0.2}Br_{0.6}Cl_{0.2})_3$ for a range of k-weights and minimum k-values, which generated the error bar for this composition in **Figure 4e**.

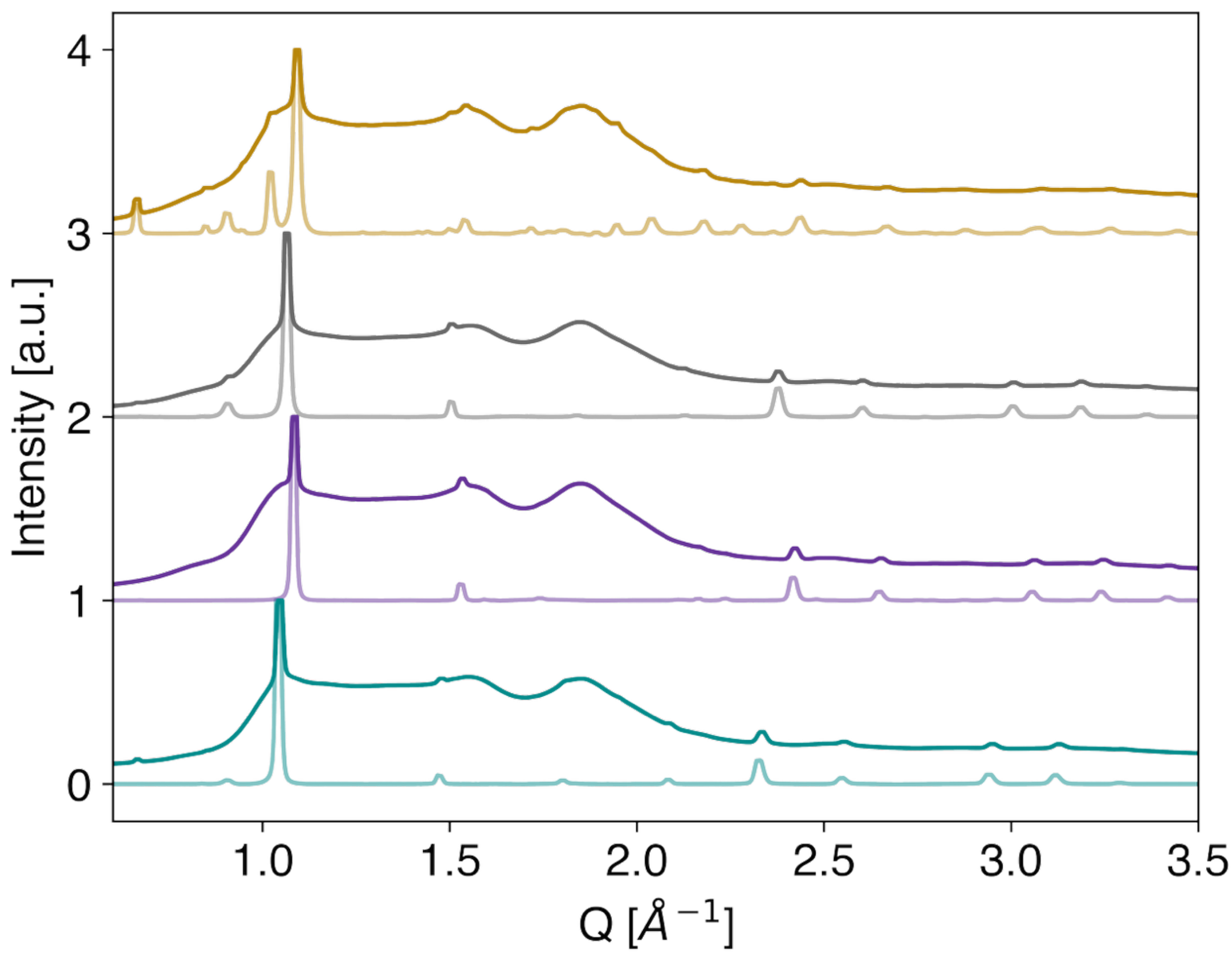

Figure S12. X-ray diffraction of mixed-halide perovskites on polyimide substrate (used for EXAFS measurements, dark) vs. glass (light; same data as **Figure 1**). The broad amorphous background results from the polyimide substrate.

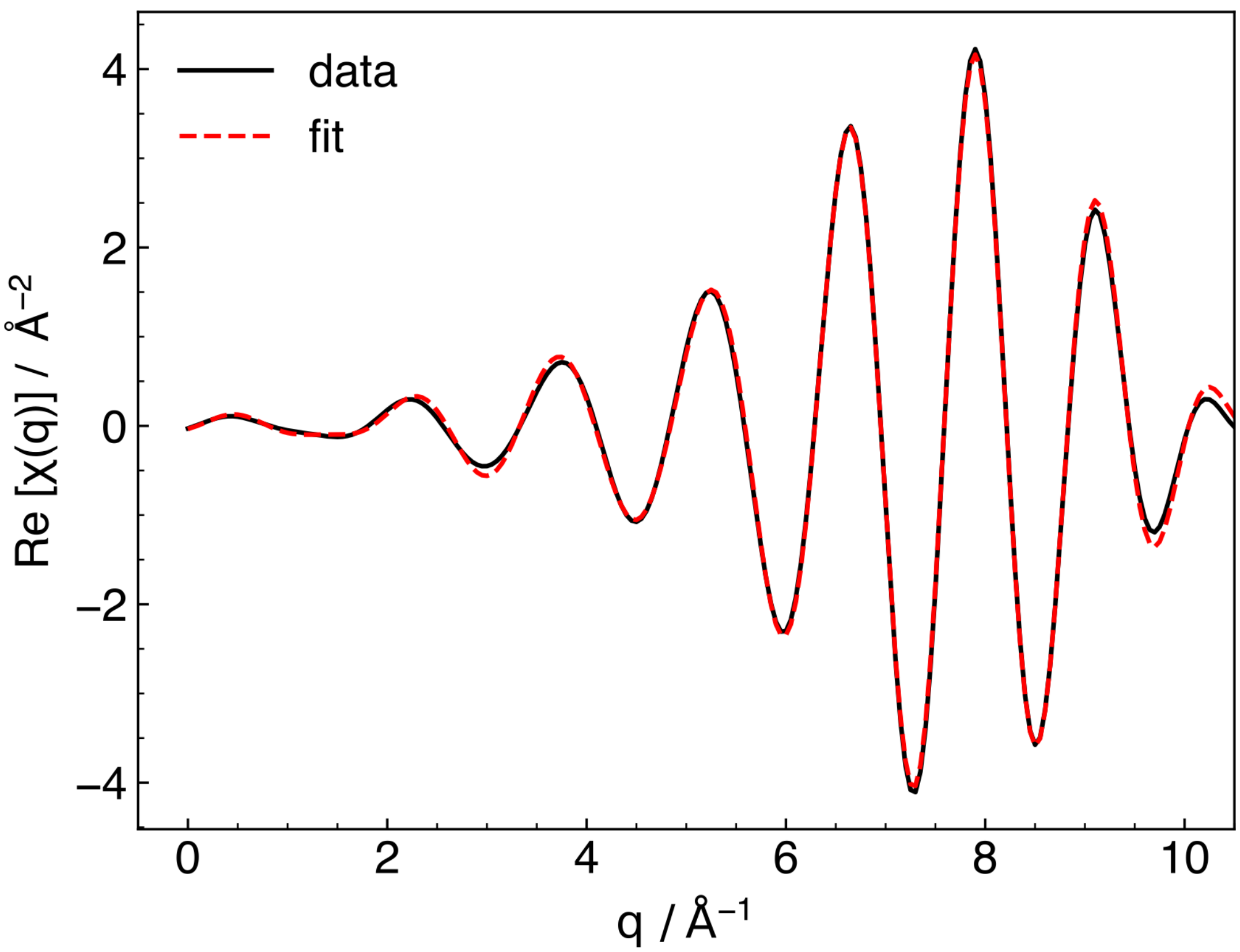

Figure S13. First-shell EXAFS fit (performed in back Fourier-transformed q-space) of $MAPb(Br_{0.6}Cl_{0.4})_3$ overlaid with the q-space data for $MAPb(Br_{0.6}Cl_{0.4})_3$. Fit values are in **Table S3**.

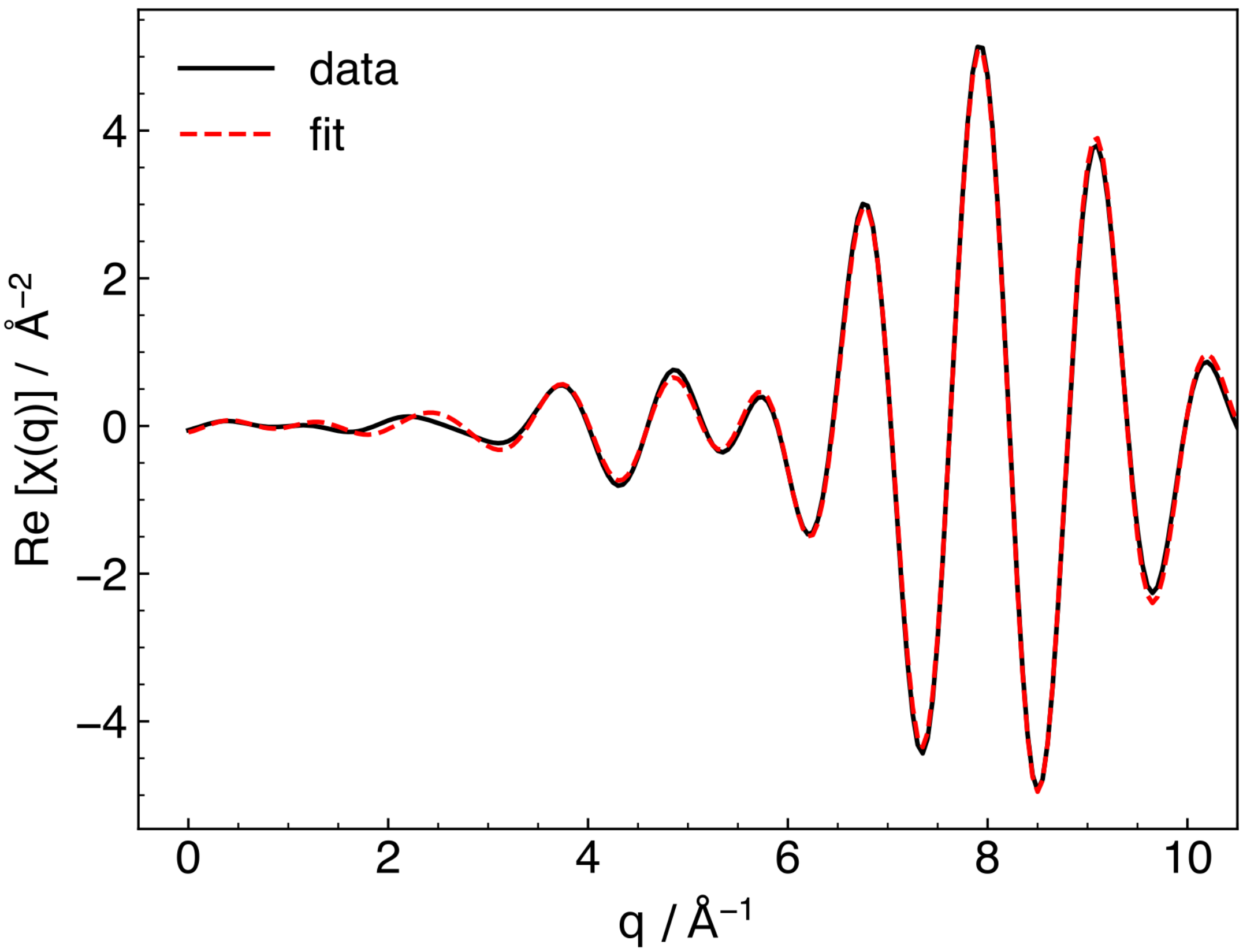

Figure S14. First-shell EXAFS fit (performed in back Fourier-transformed q-space) of $MAPb(Br_{0.6}I_{0.4})_3$ overlaid with the q-space data for $MAPb(Br_{0.6}I_{0.4})_3$. Fit values are in **Table S3**.

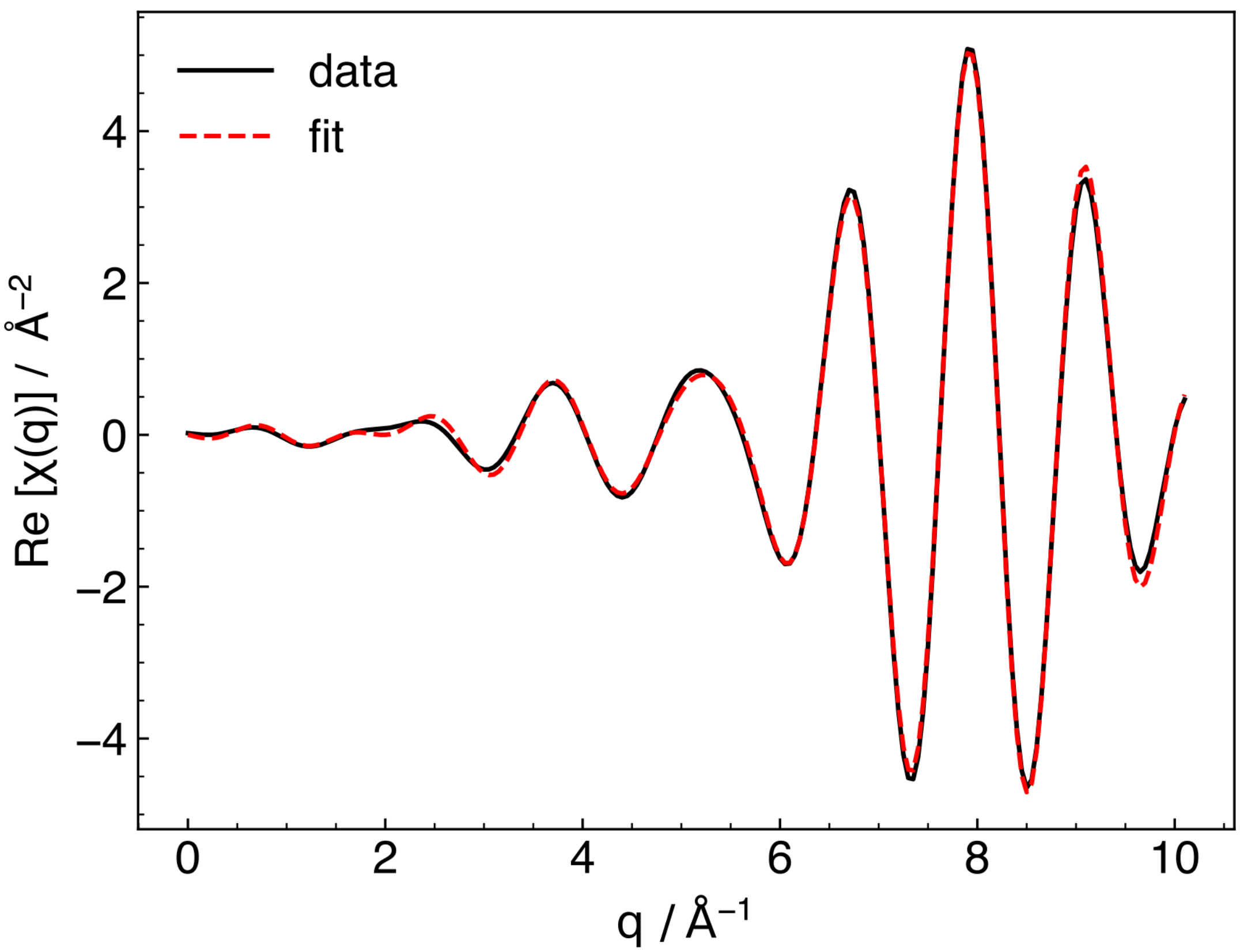

Figure S15. First-shell EXAFS fit (performed in back Fourier-transformed q-space) of $MAPb(I_{0.2}Br_{0.6}Cl_{0.2})_3$ overlaid with the q-space data for $MAPb(I_{0.2}Br_{0.6}Cl_{0.2})_3$. Fit values are in **Table S3**.

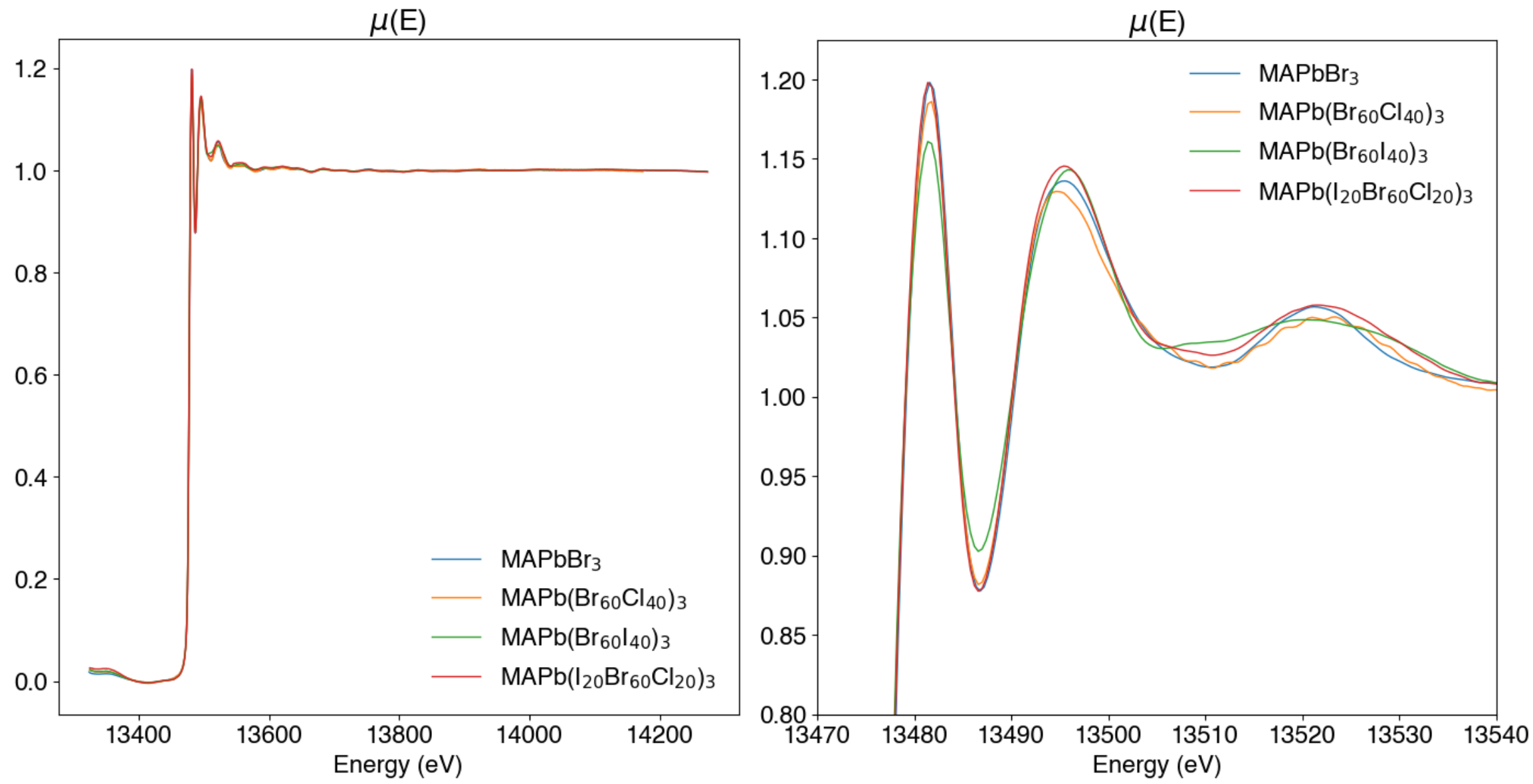

Figure S16: Full μ(E) (left) and XANES portion (right) taken at the Br K edge for the mixed-halide compositions.

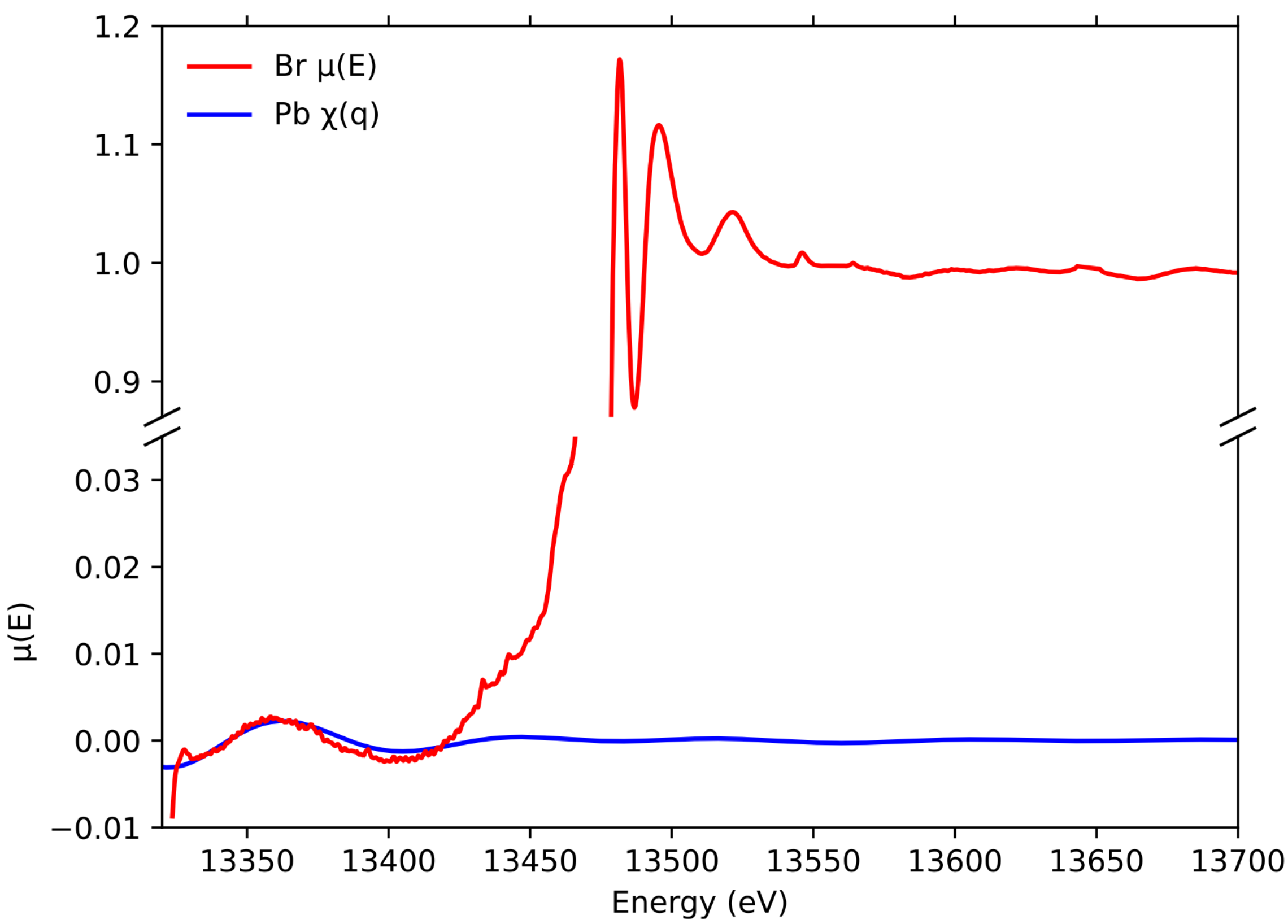

Figure S17. Overlap of the Pb L_3-edge oscillations with the Br K-edge for $MAPbBr_3$.

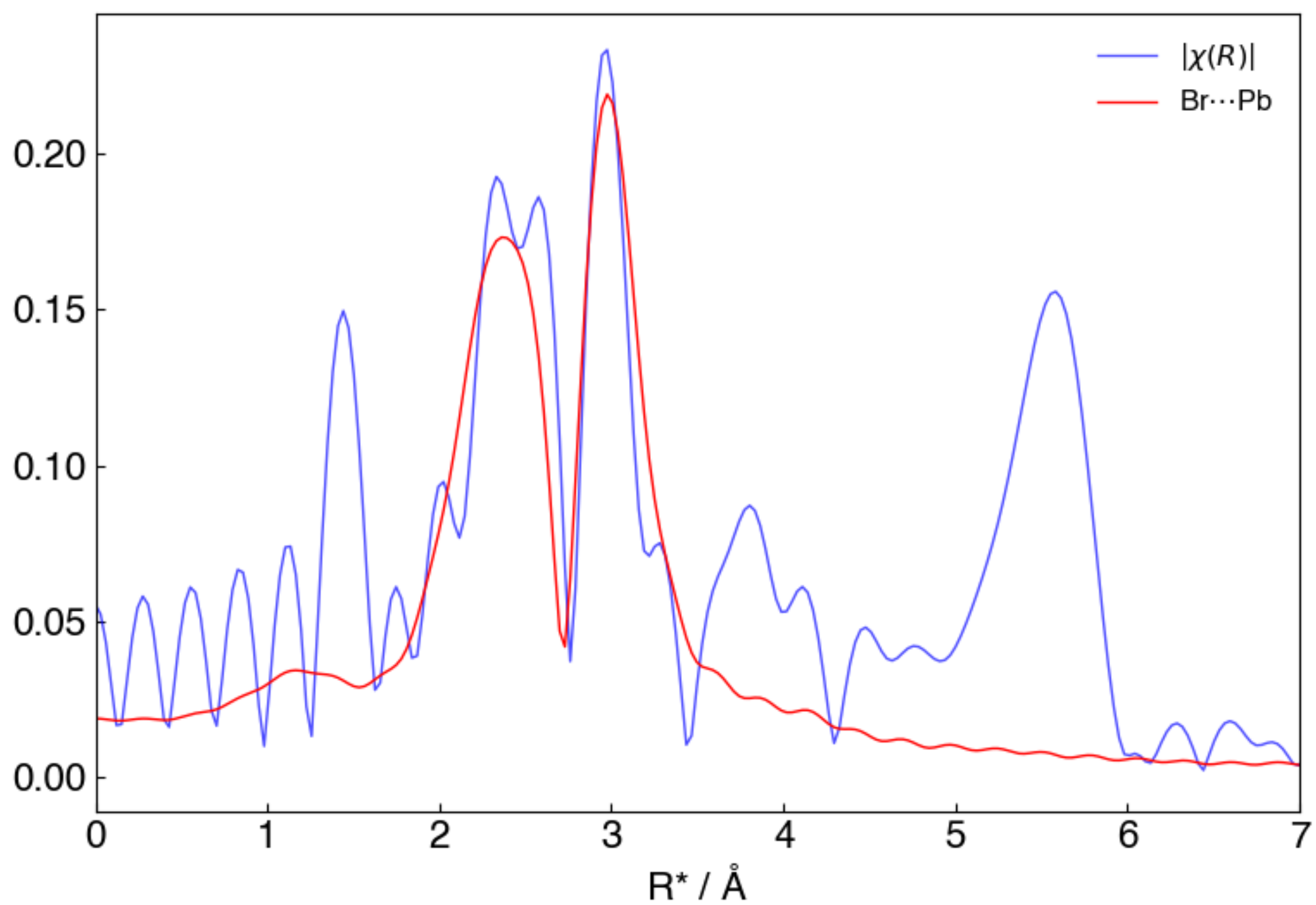

Figure S18: Magnitude of the Fourier-transformed Br K-edge EXAFS, ($|\chi(R)|$), for $MAPbBr_3$ (blue), overlaid with the FEFF-calculated first-shell, single-scattering path Br $\cdots$Pb path (red) with bond distance fixed to the orthorhombic $MAPbBr_3$ crystal structure and σ^2 fitted to the first-shell peak. The double-peaked structure at R*~2-3.5 Å is reproduced by this path alone.

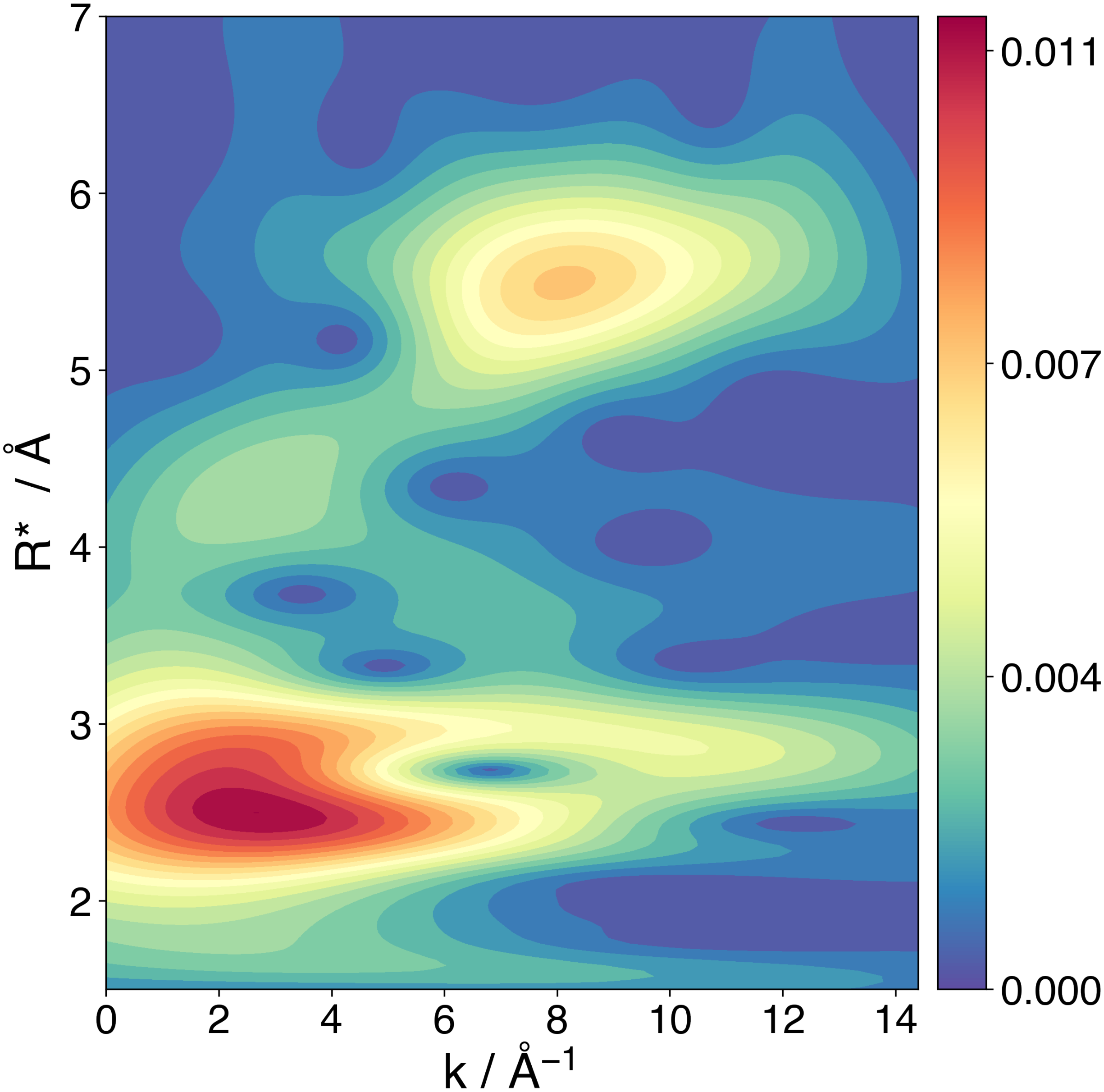

Figure S19. Full Cauchy wavelet transform (WT) for $MAPbBr_3$ measured at Br K edge.

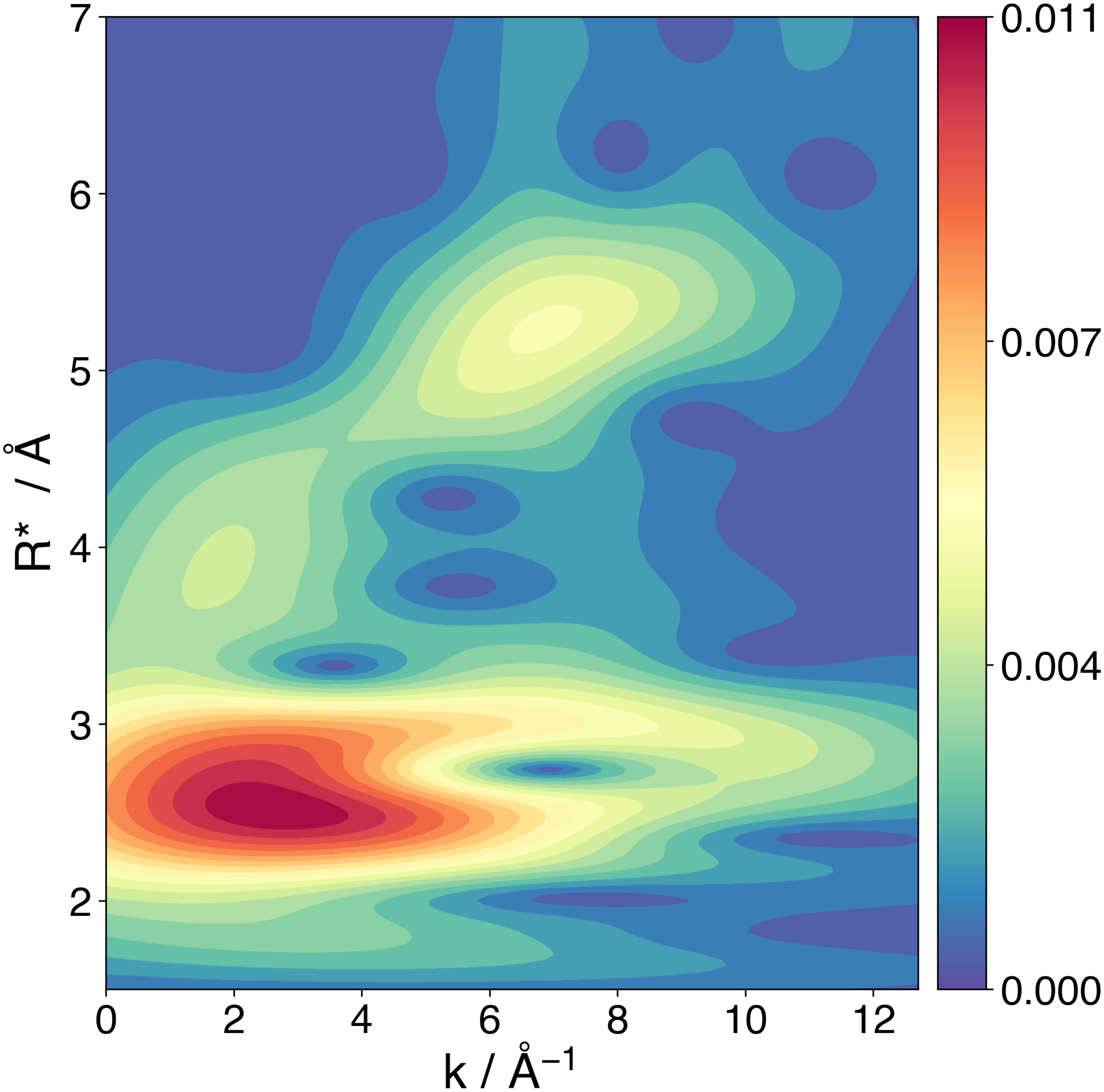

Figure S20. Full Cauchy wavelet transform for $MAPb(Br_{0.6}Cl_{0.4})_3$ measured at Br K edge.

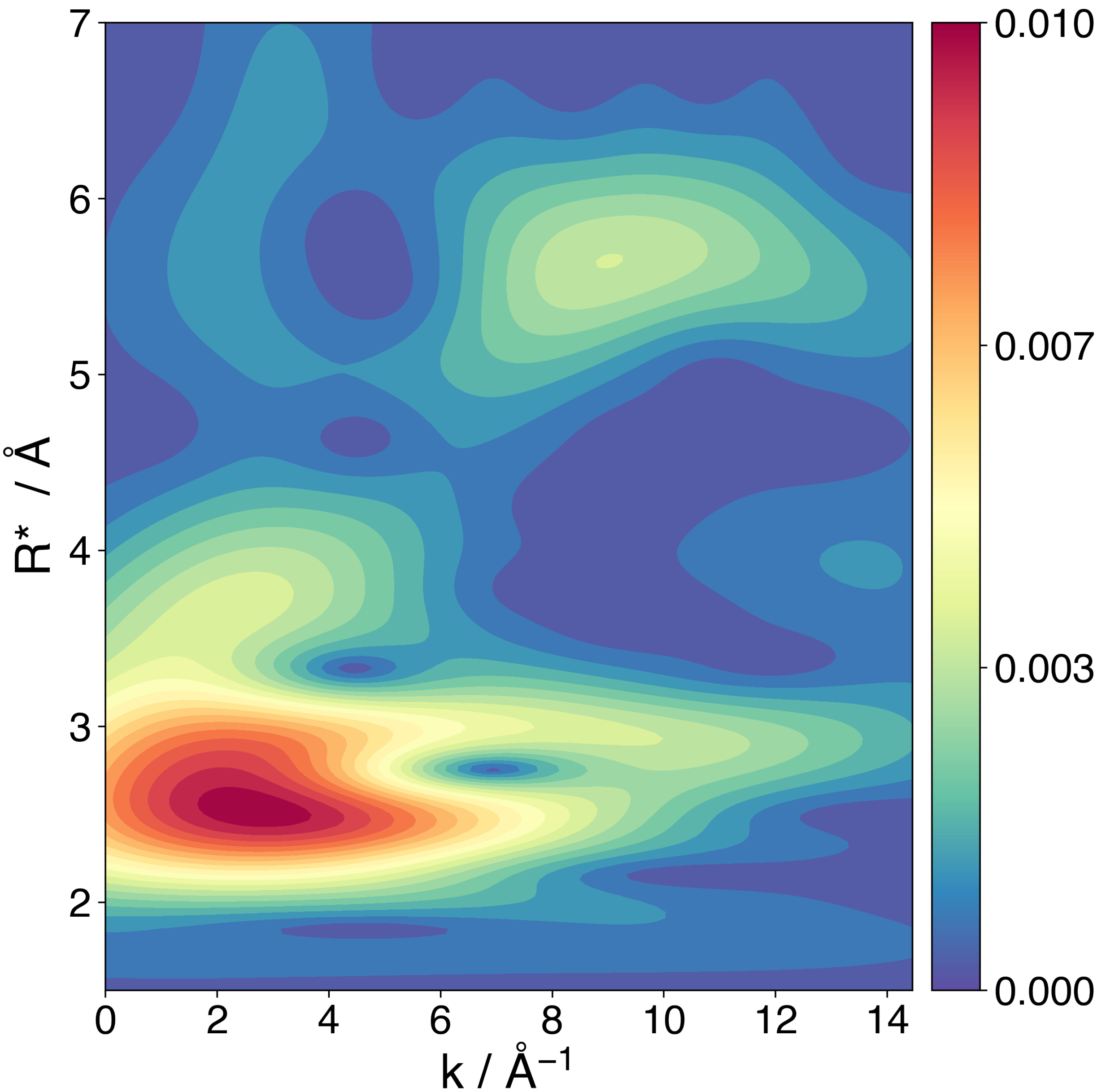

Figure S21. Full Cauchy wavelet transform for $MAPb(Br_{0.6}I_{0.4})_3$ measured at Br K edge.

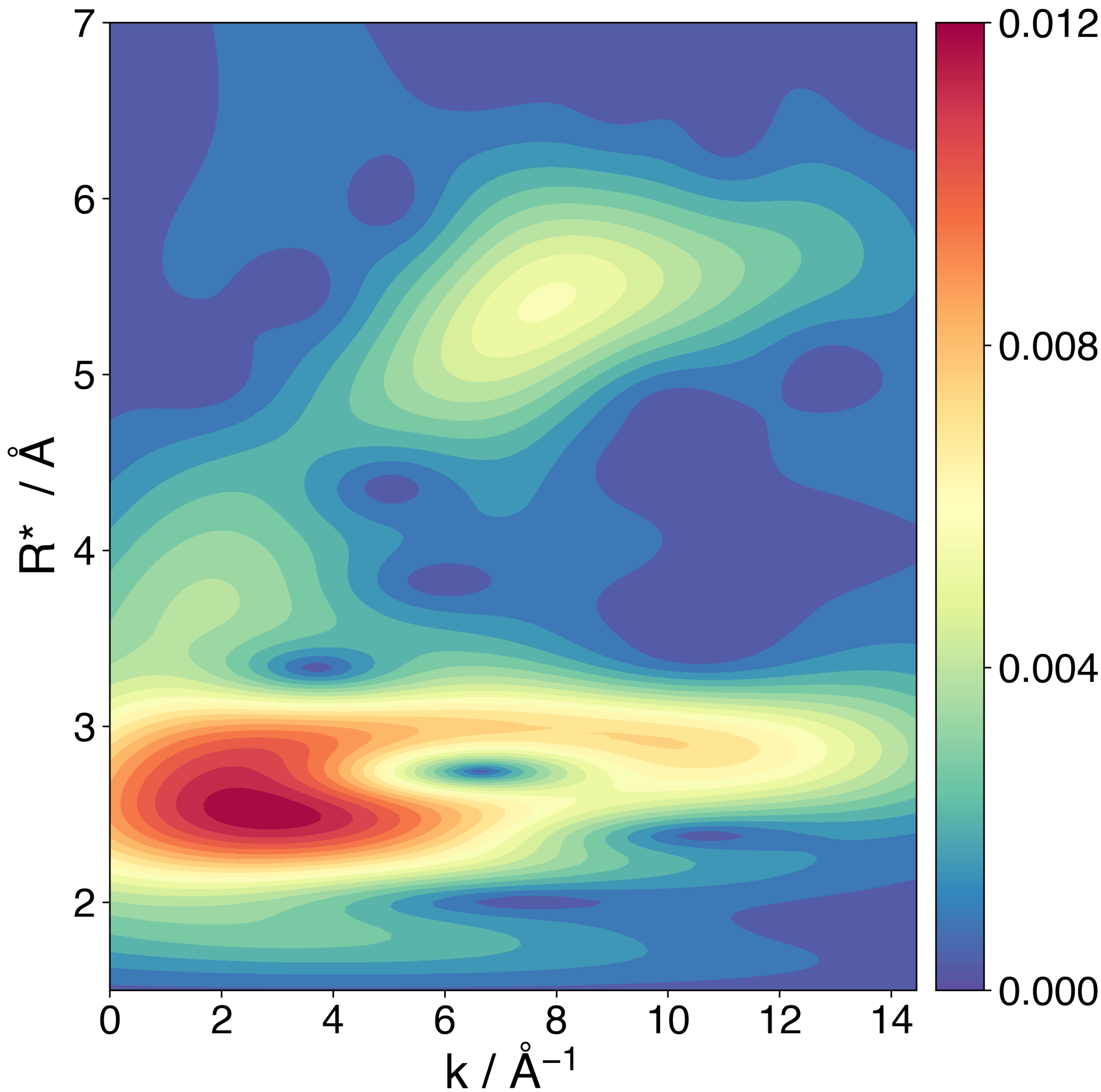

Figure S22. Full Cauchy wavelet transform for $MAPb(I_{0.2}Br_{0.6}Cl_{0.2})_3$ measured at Br K edge.

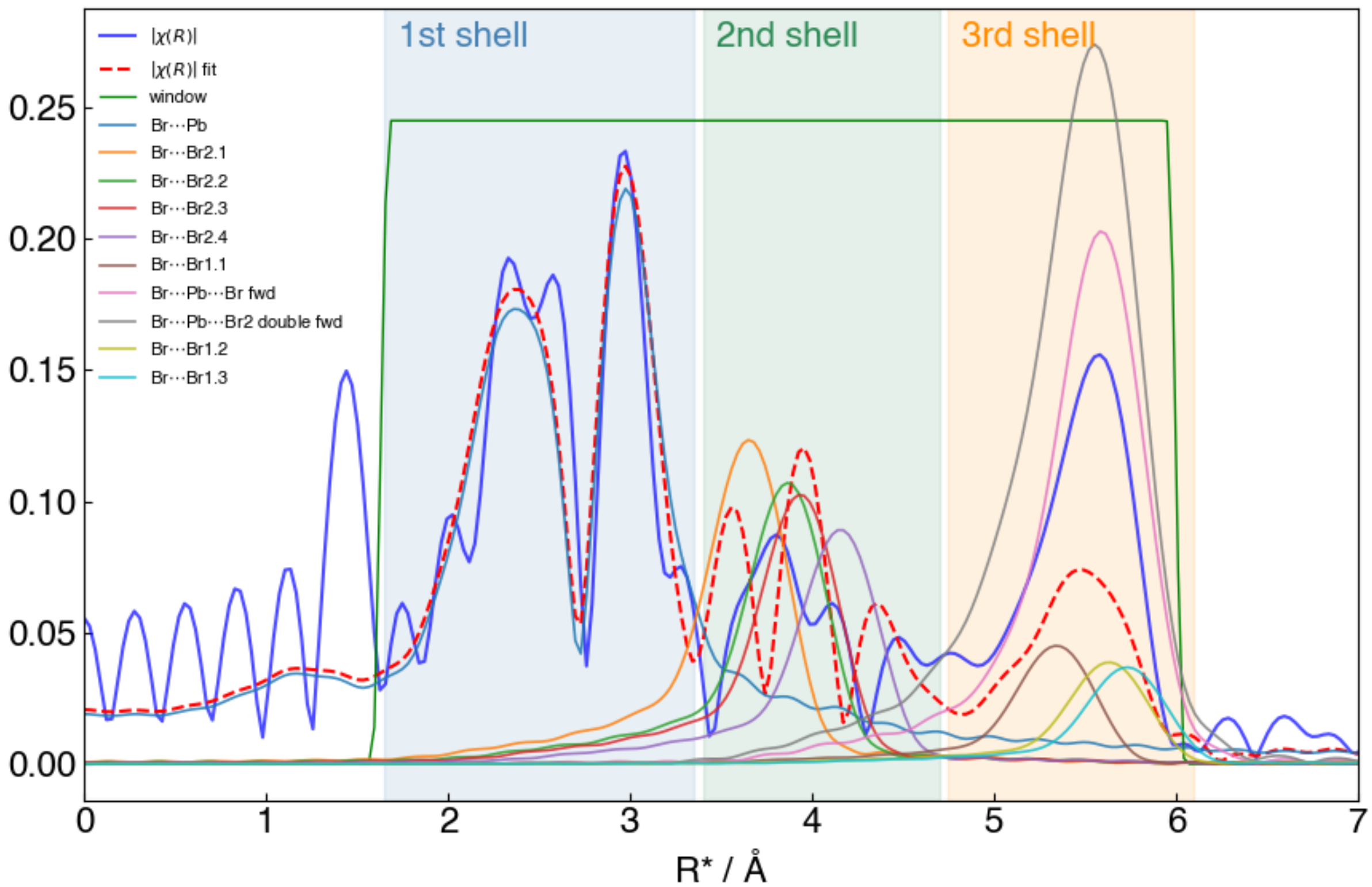

Figure S23: Magnitude of the Fourier-transformed Br K-edge EXAFS, $|\chi(R)|$, for $MAPbBr_3$ (blue), overlaid with the total fit (red dashed) and individual FEFF-calculated scattering path contributions shown. σ^2 for all paths was fixed to the same value used for the first-shell path (in Fig. S18).

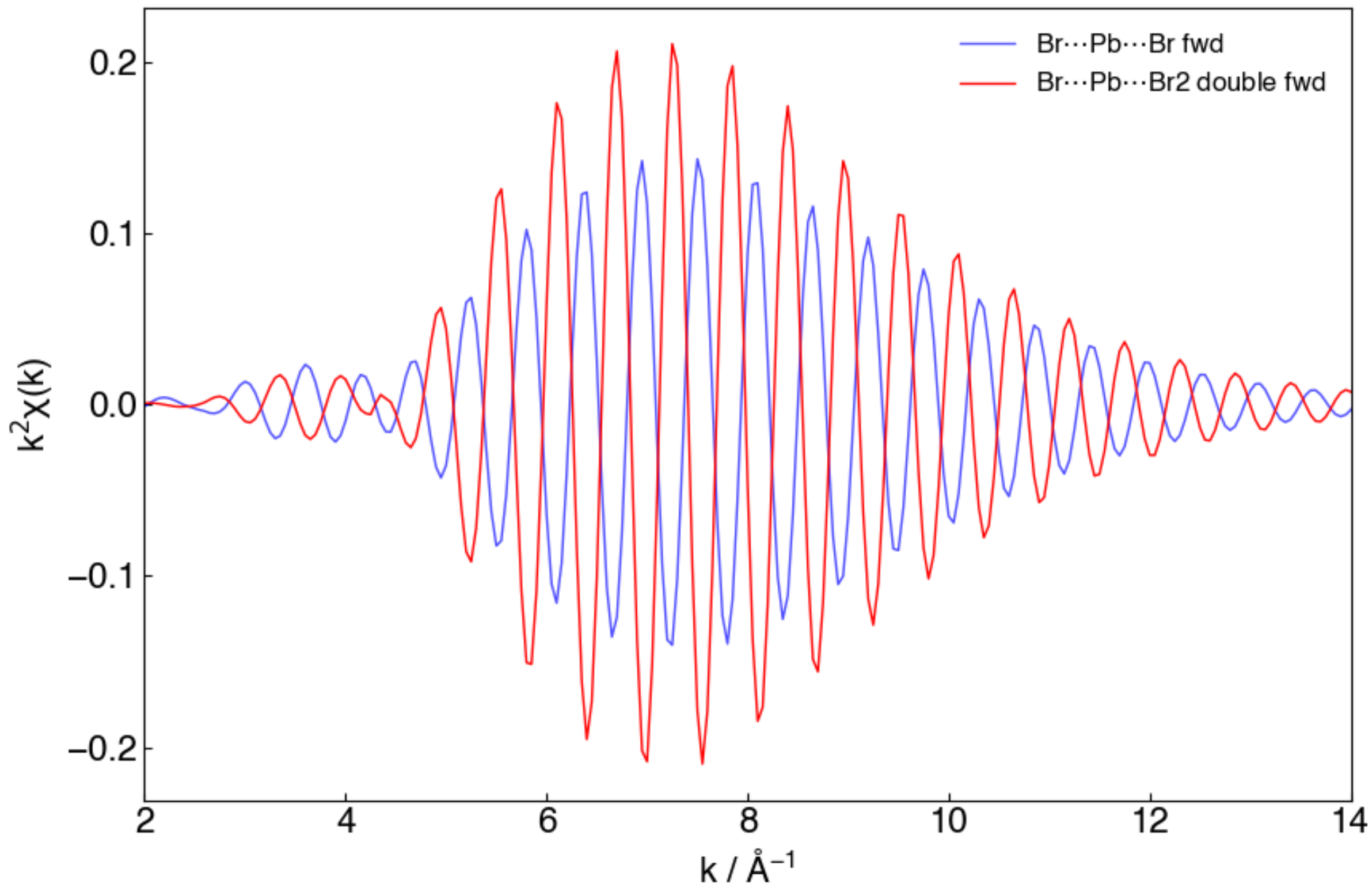

Figure S24: FEFF-calculated k^2-weighted EXAFS contributions in k -space for the two dominant third-shell multiple-scattering paths in orthorhombic $MAPbBr_3$: the single forward-scattering path Br ··· Pb ··· Br (blue) and the double forward-scattering path Br ··· Pb ··· Br ··· Pb ··· Br (red).

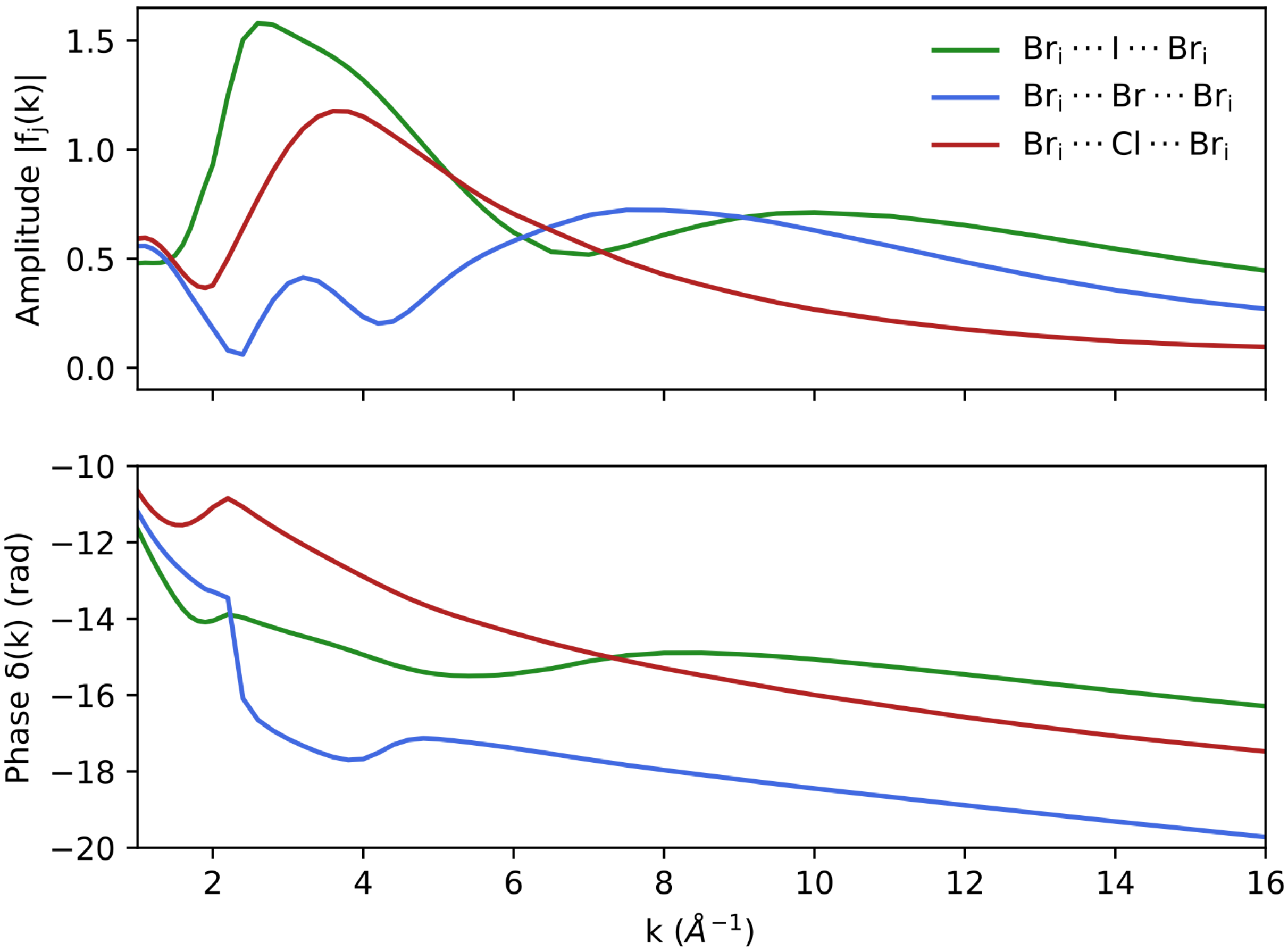

Figure S25. Calculated scattering amplitudes and phase shifts for Br-X single scattering paths as calculated in FEFF, using a Br-X distance of 3.96 Å corresponding to a $MAPbBr_3$ orthorhombic structure and changing the identity of X.

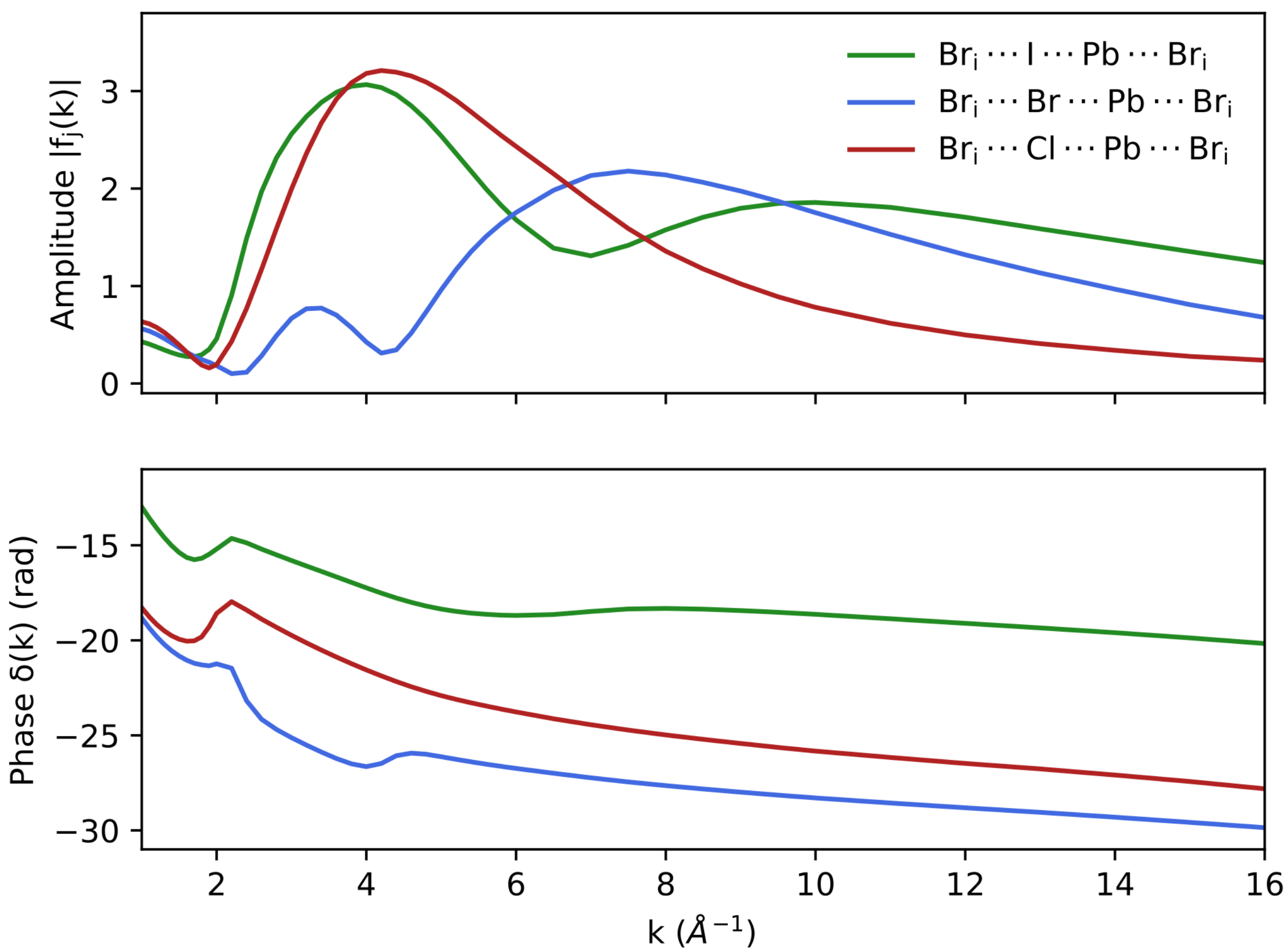

Figure S26. Calculated scattering amplitudes and phase shifts for Br_i-X-Pb-Br_i single forward scattering paths as calculated in FEFF, using a $MAPbBr_3$ orthorhombic structure and changing the identity of X. Above approximately $k = 8$ Å^{-1}, the paths for X = Br and X = I differ in phase by approximately 3π. Near $k = 7$ Å^{-1}, the paths for X = I and X = Cl differ in phase by approximately 2π.

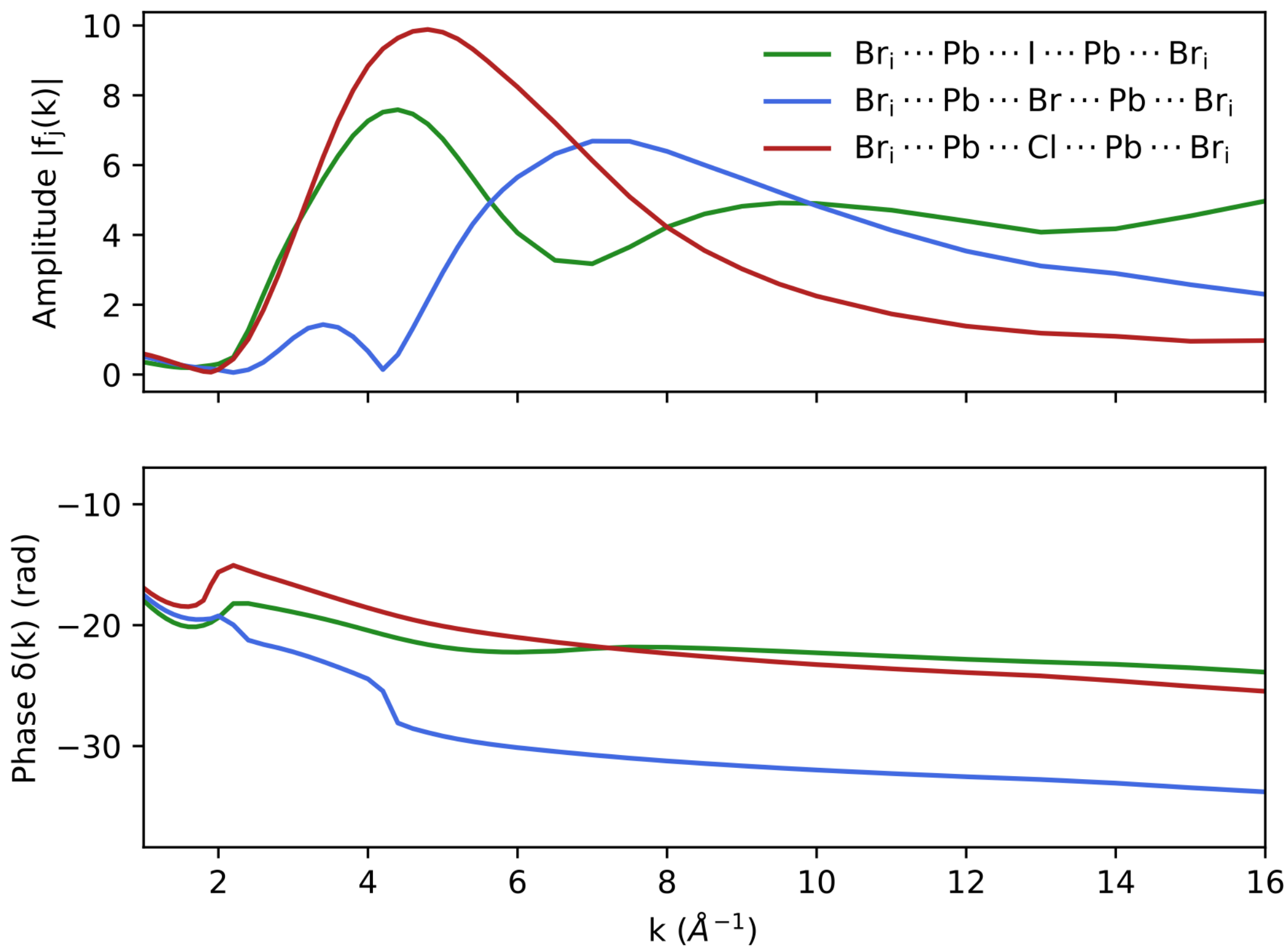

Figure S27. Calculated scattering amplitudes and phase shifts for Br_i-Pb-X-Pb-Br_i double forward scattering paths as calculated in FEFF, using a $MAPbBr_3$ orthorhombic structure and changing the identity of X. Above approximately $k = 7$ $Å^{-1}$, there is a phase difference between the X = Br path and the paths for X = Cl and X = I of approximately 3π.

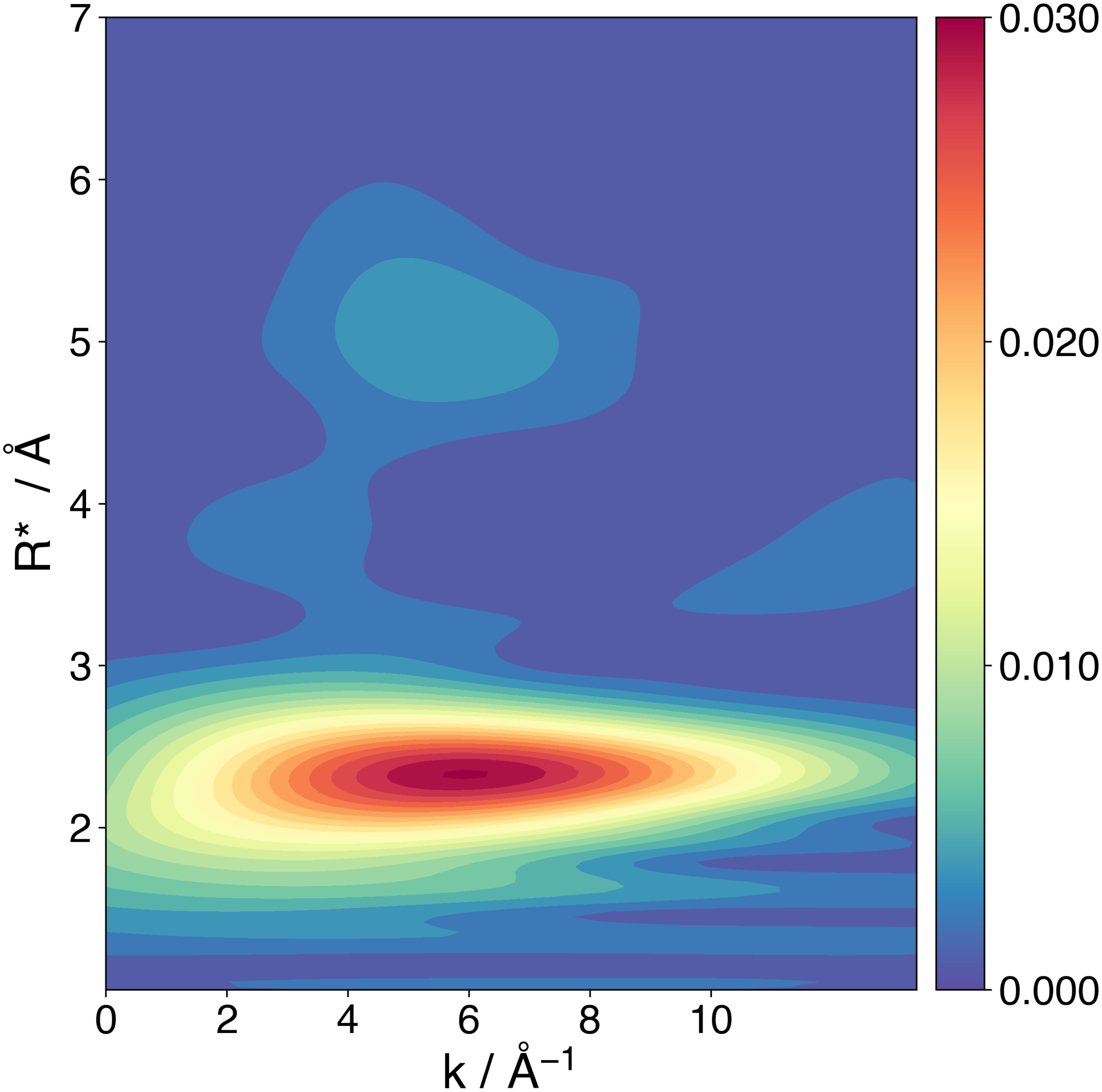

Figure S28. Full Cauchy WT of Pb L_3-edge EXAFS for $MAPbCl_3$.

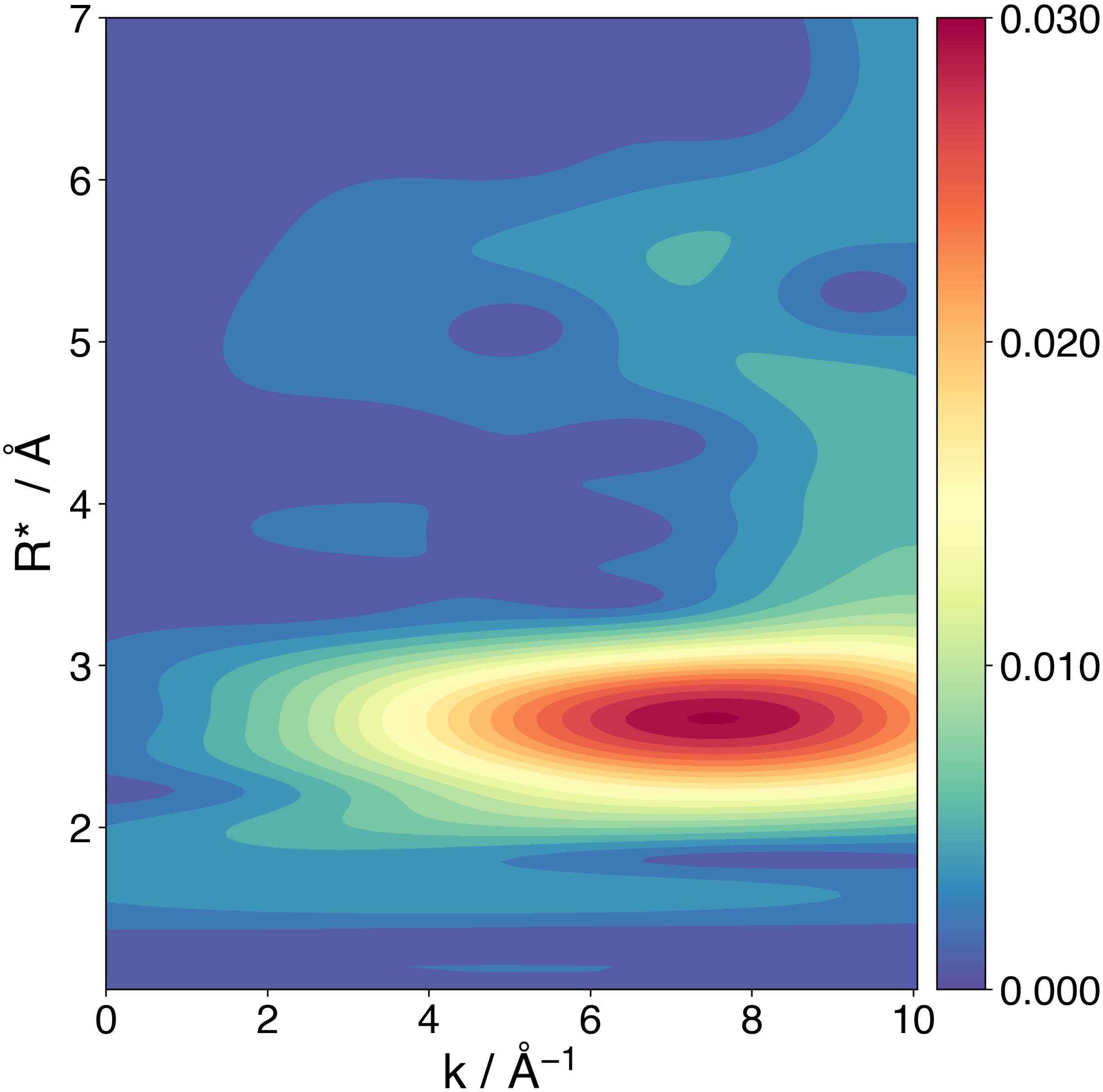

Figure S29. Full Cauchy WT of Pb L_3-edge EXAFS for $MAPbBr_3$.

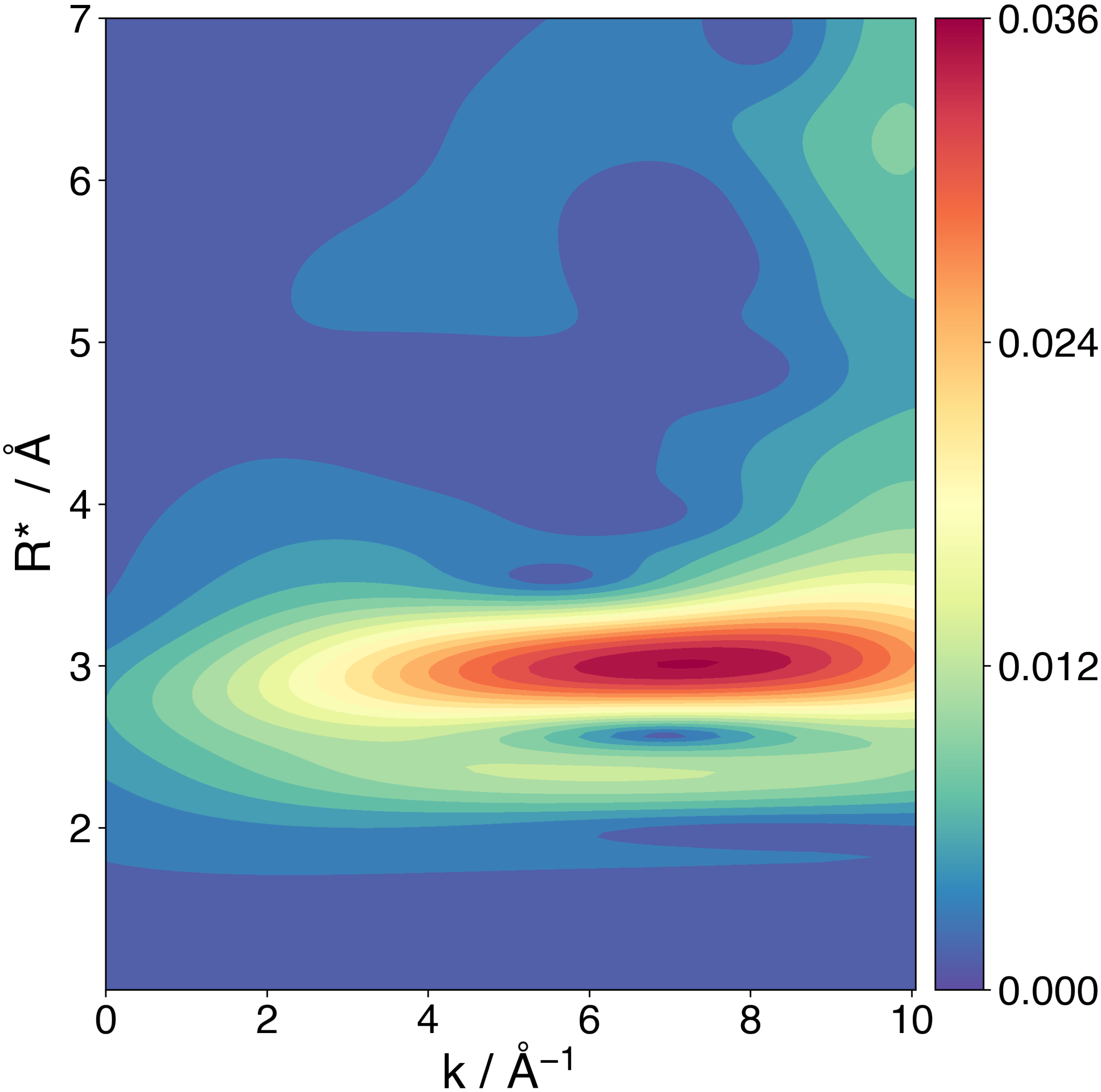

Figure S30. Full Cauchy WT of Pb L_3-edge EXAFS for $MAPbI_3$.

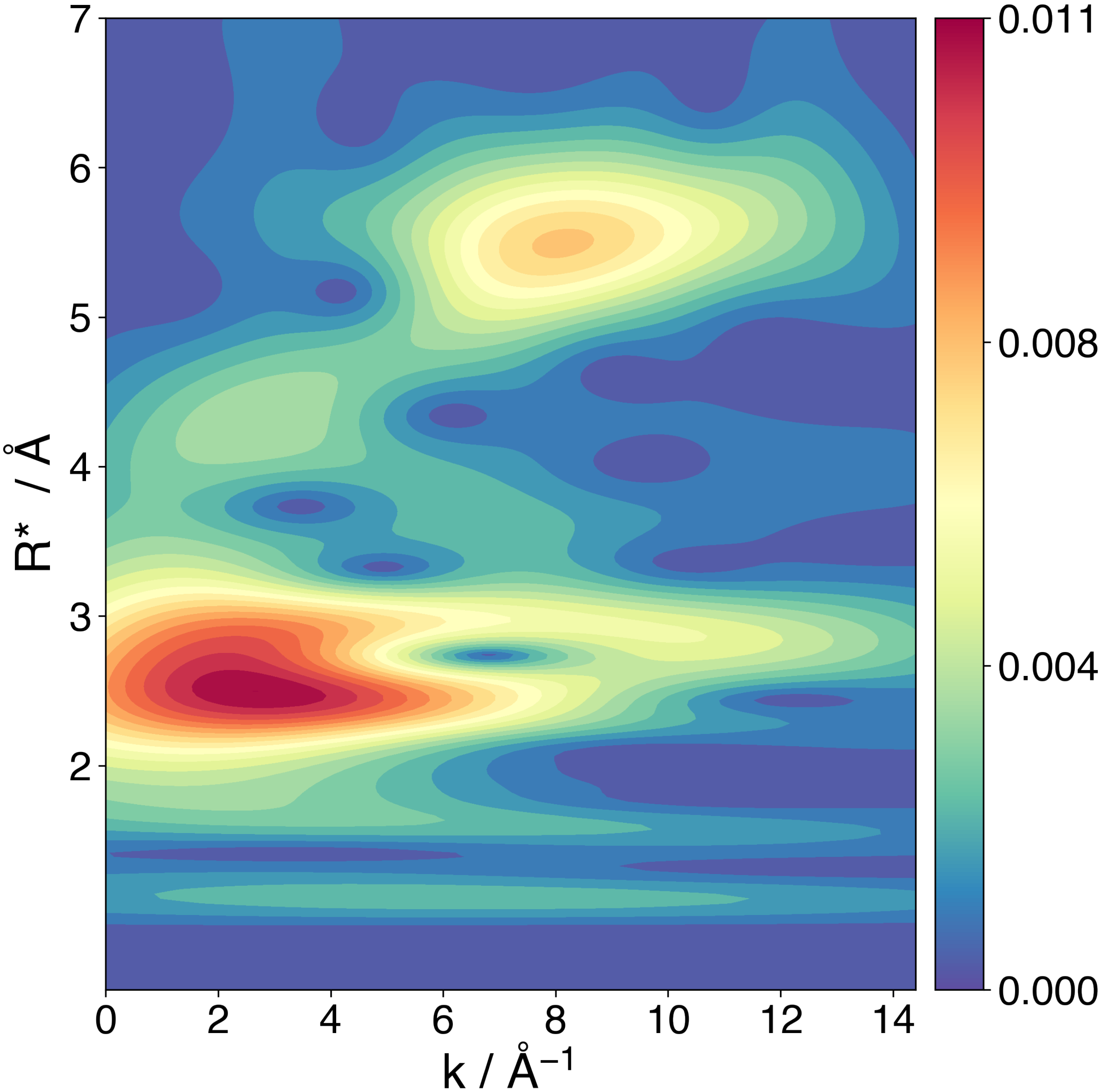

Figure S31. Full Cauchy WT of Br K-edge EXAFS for $MAPbBr_3$, computed using r_{bkg} = 1.0 Å.

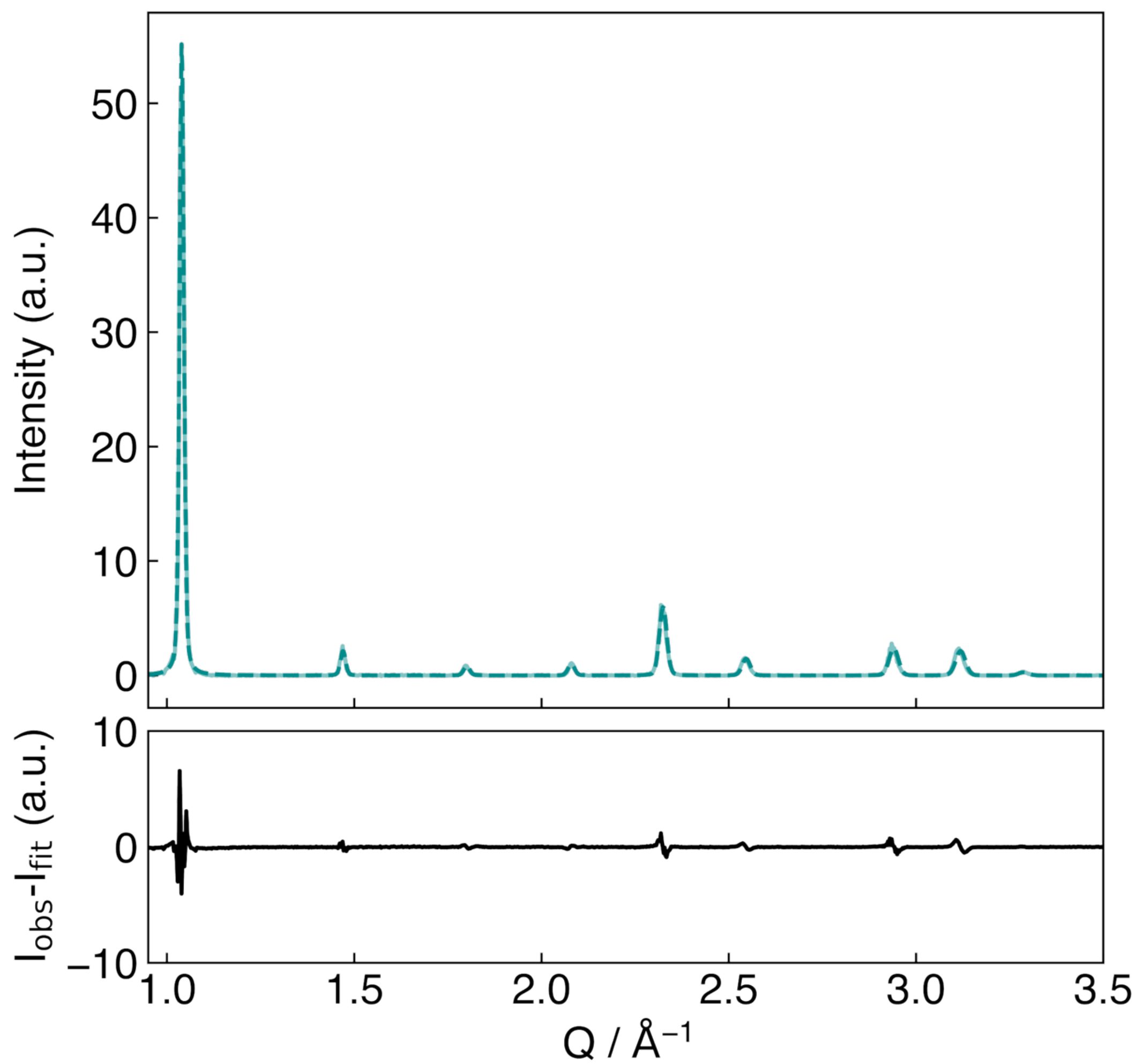

Figure S32. Integrated X-ray scattering of $MAPb(Br_{0.6}I_{0.4})_3$ (solid line) and calculated scattering from Pawley fit (dashed line). Peak positions are well described by a cubic $Pm\bar{3}m$ model, with $a = 6.04$ Å .

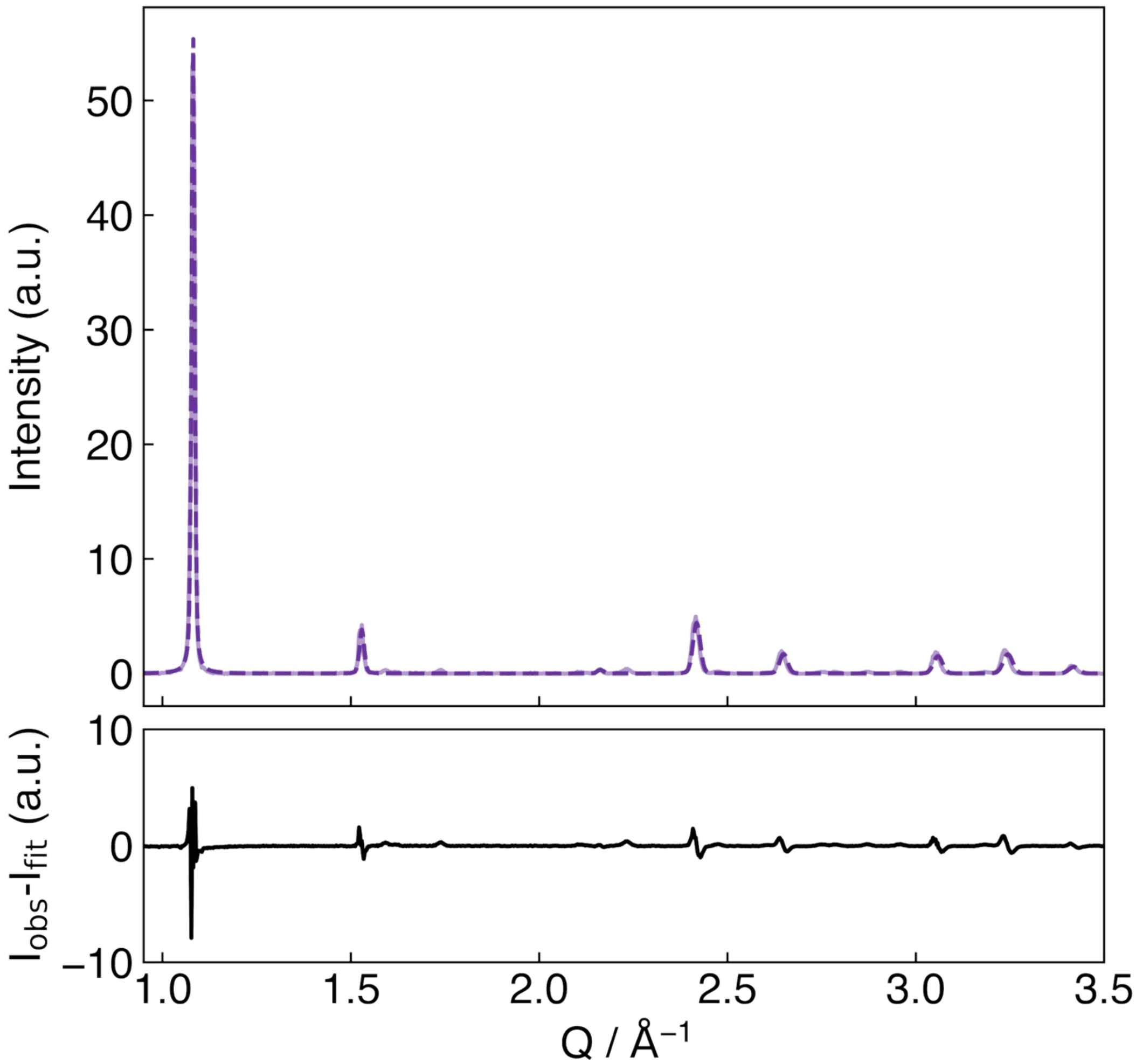

Figure S32. Integrated X-ray scattering of $MAPb(Br_{0.6}Cl_{0.4})_3$ (solid line) and calculated scattering from Pawley fit (dashed line). Peak positions are well described by a cubic $Pm\bar{3}m$ model, with $a = 5.81$ Å . Reflections not captured by fit consistent with trace crystalline $PbCl_2$

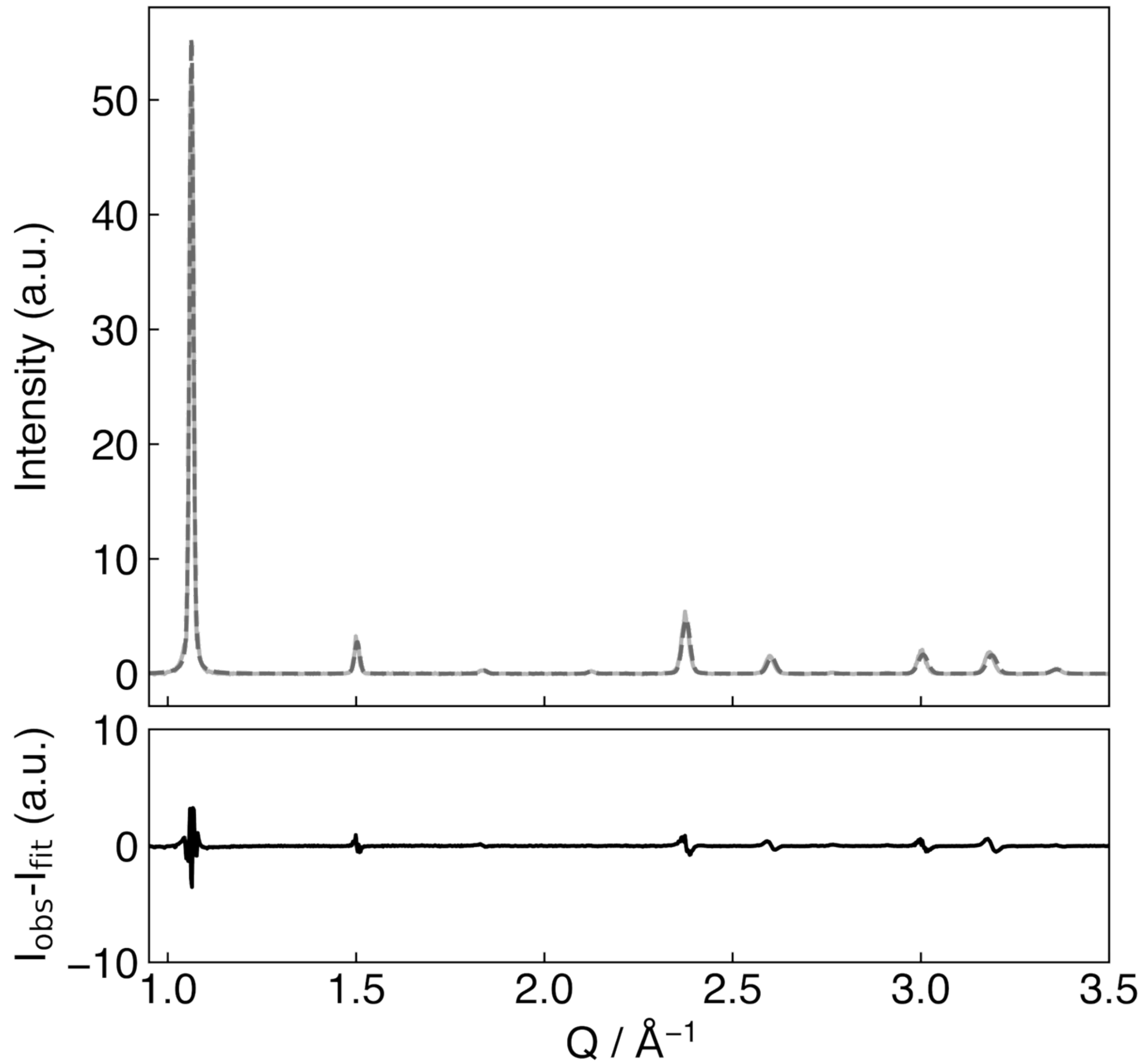

Figure S33. Integrated X-ray scattering of $MAPb(Br_{0.6}I_{0.2}Cl_{0.2})_3$ (solid line) and calculated scattering from Pawley fit (dashed line). Peak positions are well described by a cubic $Pm\bar{3}m$ model, with $a = 5.91$ Å .

Nominal composition	Goldschmidt tolerance factor, t	Octahedral factor, μ	Ionic Pb-X distance (Å)	Fitted EXAFS Pb-X (Å)
$MAPbI_3$	0.912	0.541	3.39	3.18
$MAPbBr_3$	0.927	0.607	3.15	2.98
$MAPbCl_3$	0.938	0.657	3.00	2.78-3.017
$MAPb(Br_{0.6}Cl_{0.4})_3$	0.931	0.626	N/A	2.844, 2.962
$MAPb(Br_{0.6}I_{0.4})_3$	0.921	0.579	N/A	2.979, 3.156
$MAPb(I_{0.2}Br_{0.6}Cl_{0.2})_3$	0.926	0.602	N/A	2.847- 3.174
$MAPb(I_{0.4}Br_{0.2}Cl_{0.4})_3$	0.925	0.596	N/A	N/A

Table S11. Calculated Goldschmidt tolerance factor and octahedral factors for the nominal compositions studied in this work, calculated using the Shannon ionic radii (Pb^{2+}: 1.19 Å; I^-: 2.20

Å; Br^-: 1.96 Å; Cl^-: 1.81 Å)[7] and effective R_{MA} = 2.17 Å,[8] along with comparison with ionic Pb-X distance and fitted Pb-X bond lengths.

References

(1) Ashiotis, G.; Deschildre, A.; Nawaz, Z.; Wright, J. P.; Karkoulis, D.; Picca, F. E.; Kieffer, J. The Fast Azimuthal Integration Python Library: pyFAI. *J. Appl. Crystallogr.* **2015**, *48* (2), 510–519. https://doi.org/10.1107/S1600576715004306.

(2) Dane, T. Tgdane/Pygix, 2024. https://github.com/tgdane/pygix (accessed 2026-04-12).

(3) Toby, B. H.; Von Dreele, R. B. GSAS-II: The Genesis of a Modern Open-Source All Purpose Crystallography Software Package. *J. Appl. Crystallogr.* **2013**, *46* (2), 544–549. https://doi.org/10.1107/S0021889813003531.

(4) Weller, M. T.; Weber, O. J.; Henry, P. F.; Pumpo, A. M. D.; Hansen, T. C. Complete Structure and Cation Orientation in the Perovskite Photovoltaic Methylammonium Lead Iodide between 100 and 352 K. *Chem. Commun.* **2015**, *51* (20), 4180–4183. https://doi.org/10.1039/C4CC09944C.

(5) Swainson, I. P.; Hammond, R. P.; Soullière, C.; Knop, O.; Massa, W. Phase Transitions in the Perovskite Methylammonium Lead Bromide, CH3ND3PbBr3. *J. Solid State Chem.* **2003**, *176* (1), 97–104. https://doi.org/10.1016/S0022-4596(03)00352-9.

(6) Chi, L.; Swainson, I.; Cranswick, L.; Her, J.-H.; Stephens, P.; Knop, O. The Ordered Phase of Methylammonium Lead Chloride CH3ND3PbCl3. *J. Solid State Chem.* **2005**, *178* (5), 1376–1385. https://doi.org/10.1016/j.jssc.2004.12.037.

(7) Shannon, R. D. Revised Effective Ionic Radii and Systematic Studies of Interatomic Distances in Halides and Chalcogenides. *Acta Crystallogr. Sect. A* **1976**, *32* (5), 751–767. https://doi.org/10.1107/S0567739476001551.

(8) Kieslich, G.; Sun, S.; Cheetham, A. K. An Extended Tolerance Factor Approach for Organic–Inorganic Perovskites. *Chem. Sci.* **2015**, *6* (6), 3430–3433. https://doi.org/10.1039/c5sc00961h.
